# Clinical presentation, investigation findings, and outcomes of hypophysitis in sarcoidosis: a systematic review

**DOI:** 10.1007/s11102-026-01753-y

**Published:** 2026-08-20

**Authors:** Julia F B Cavalcanti, Sara Caixeta de Souza, Leandro Tavares Lucato, Andrea Glezer, Guilherme Diogo Silva

**Affiliations:** 1 Department of Neurology, Hospital das Clinicas, University of São Paulo, Doutor Enéas de Carvalho Aguiar, 255, São Paulo, Brazil; 2 Department of Radiology, Hospital das Clinicas, University of São Paulo, São Paulo, Brazil; 3 Unit of Neuroendocrinology, Division of Endocrinology and Metabolism, Hospital das Clinicas, University of São Paulo, São Paulo, Brazil; 4 Medical Research Laboratory 15 (LIM-15), University of São Paulo Medical School, São Paulo, Brazil

**Keywords:** Sarcoidosis, Neurosarcoidosis, Hypophysitis, Pituitary, Pituitary disease, Hypopituitarism

## Abstract

**Purpose:**

Hypophysitis in sarcoidosis is a rare manifestation of neurosarcoidosis that may cause permanent endocrine dysfunction, visual impairment, and neurological morbidity. We performed a systematic review to characterize its clinical presentation, diagnostic findings, treatment, and outcomes.

**Methods:**

PubMed, Embase, and Scopus were searched from inception to January 22, 2025, for reports of adult patients with sarcoidosis-related hypophysitis. Cases were reassessed according to the 2018 Neurosarcoidosis Consortium Consensus Group criteria. Individual patient data were extracted regarding clinical manifestations, hormonal abnormalities, imaging findings, systemic involvement, treatment, recurrence, and long-term outcomes. Sensitivity analyses restricted the cohort to patients meeting definite or probable criteria and stratified cases by publication era (1954–1999, 2000–2017, 2018–2024) to assess the robustness of findings and the impact of temporal heterogeneity.

**Results:**

A total of 166 studies comprising 274 patients were included. Diagnostic certainty was definite in 26.3%, probable in 47.8%, and possible in 25.9% of cases. Median age was 37 years (IQR 28–48), and 53.5% were male. AVP deficiency was the most frequent manifestation (53.6%), followed by gonadal dysfunction (38.3%), visual loss (36.1%), headache (32.2%), and fatigue (30.5%). Central hypogonadism was the most common anterior pituitary abnormality (74.1%), followed by central hypothyroidism (63.6%) and hyperprolactinemia (58.1%). MRI most commonly demonstrated pituitary stalk thickening (43.1%) and sellar/suprasellar masses (36.4%). CSF analysis frequently showed elevated protein (68.9%) and pleocytosis (59.5%). Systemic involvement occurred in 80.3% of patients, predominantly affecting lymph nodes and lungs. Although corticosteroids were the mainstay of treatment, recurrence occurred in 43.1% of patients, chronic hormone replacement was required in 36.2%, and long-term desmopressin dependence occurred in 30.5%. Findings were materially unchanged when restricted to the definite/probable cohort (*n* = 203) and across publication eras, despite increased MRI use and greater adoption of steroid-sparing and biologic therapies over time.

**Conclusions:**

Hypophysitis in sarcoidosis should be suspected in patients presenting with stalk thickening, sellar lesions, AVP deficiency, or unexplained hypogonadism. Because serum and CSF biomarkers showed limited sensitivity, systemic investigation and extracranial biopsy are essential. Despite treatment, recurrence and persistent endocrine dysfunction remain common, highlighting the need for long-term multidisciplinary follow-up.

**Supplementary Information:**

The online version contains supplementary material available at https://doi.org/10.1007/s11102-026-01753-y.

## Introduction

 Sarcoidosis is a systemic granulomatous disease of unknown etiology [1–3], histopathologically defined by the presence of noncaseating epithelioid cell granulomas [2, 3]. Neurological involvement, termed neurosarcoidosis (NS), occurs in 5% to 15% of patients [1, 2, 4, 5], though subclinical involvement may reach 25% in autopsy and imaging studies [3]. Among its manifestations, hypothalamic-pituitary (HP) involvement is particularly clinically significant, as it may result in profound, often irreversible endocrine dysfunction. Involvement of adjacent structures, including the optic apparatus and neighboring cranial nerves, is also common and may additionally produce visual impairment, cranial neuropathies, and headache.

HP sarcoidosis occurs in 6–9% of NS cases [4–6], likely reflecting the predilection of sarcoid granulomas for the basal meninges [7]. Basal leptomeningeal inflammation may extend along the subarachnoid space of the pituitary infundibulum, with contiguous involvement of the pituitary gland itself, while separate parenchymal spread along Virchow-Robin perivascular spaces may account for hypothalamic infiltration [1]. It accounts for 10% to 25% of parenchymal NS cases [2], with the hypothalamic region representing the most commonly involved parenchymal site [1]. The resulting endocrine syndrome is a heterogeneous spectrum of endocrine abnormalities that often persist despite treatment [1, 6, 8] even when radiological improvement is achieved, supporting the presence of irreversible hypothalamic or pituitary injury.

Anterior pituitary dysfunction is reported more frequently than posterior pituitary involvement [1], with manifestations including gonadotropin deficiency, TSH deficiency, hyperprolactinemia, and central hypocortisolism. Central hypogonadism appears to be the most frequently reported abnormality in recent series [3, 8–10] Posterior pituitary and hypothalamic involvement typically presents with polyuria-polydipsia syndrome attributable to AVP deficiency (AVP-D), syndrome of inappropriate secretion of anti-diuretic hormone (SIADH) or hypothalamic thirst dysregulation [1, 7, 8, 11], as well as broader vegetative disturbances including appetite, temperature, sleep, libido, and weight [1, 7].

Diagnosis of NS remains particularly challenging. Clinical and radiological features often overlap with infectious, inflammatory, and neoplastic disorders, and no specific biomarker exists. The 2018 Neurosarcoidosis Consortium Consensus Group (NCCG) criteria [12] provide a standardized framework for classifying cases as possible, probable, or definite NS based on clinical, radiological, and histopathological findings.

The published literature on HP consists predominantly of small case series, single-institution retrospective studies, and individual case reports. This heterogeneity has precluded a comprehensive understanding of the condition and makes interpretation difficult for the practicing clinician. A systematic review of published cases provides an ideal framework for synthesizing available evidence and generating clinically meaningful conclusions. Accordingly, the present systematic review addresses the following questions: [1] What are the most common neurological and endocrine manifestations of sarcoidosis-related hypophysitis [2]? What are the characteristic neuroimaging, laboratory, and histopathological findings [3]? What is the typical clinical course, and what factors influence treatment response and long-term prognosis?

## Methods

We conducted a systematic review of the clinical presentation, investigation findings, treatment strategies, and clinical outcomes of hypophysitis in neurosarcoidosis. This study is compliant with the Preferred Reporting Items for Systematic Reviews and Meta-analyses (PRISMA) reporting guideline and was registered on PROSPERO (CRD420251038243).

### Search strategy

PubMed, SCOPUS, and EMBASE were systematically searched from database inception to January 22nd, 2025, using a combination of Medical Subject Headings (MeSH) and relevant keywords to identify case reports and case series of sarcoidosis-related hypophysitis. Detailed search strategies for each database are provided in the Supplementary material. In addition, reference lists of included studies were manually screened to identify further eligible articles. No language restrictions were applied.

### Study selection: inclusion criteria

Patients diagnosed with neurosarcoidosis based on tissue biopsy or complementary exams such as magnetic resonance imaging, cerebral spinal fluid analysis, and/or electromyography/nerve conduction studies, based on the 2018 NCCG criteria [12], and evidence of hypophysitis (sellar mass or pituitary stalk thickening on neuroimaging), and/or abnormalities of endocrine panel, such as GH, TSH, ACTH, FSH, LH, Prolactin, T3, T4, Estrogen, Progesterone, Testosterone, Cortisol.

Given that a substantial proportion of included studies were published before the establishment of standardized diagnostic criteria, we systematically reviewed the clinical, radiological, and ancillary investigative data reported in each case to determine whether they met current diagnostic criteria for NS. Cases were included only if sufficient information was available to support classification according to contemporary consensus definitions, and patients with possible, probable and definite NS were included in this study.

### Study selection: exclusion criteria

Studies were excluded if they included patients younger than 18 years old or if cases did not meet the predefined inclusion criteria. Specifically, reports lacking a confirmed diagnosis of neurosarcoidosis according to the 2018 NCCG were excluded.

### Study selection process

Following removal of duplicates, two reviewers (JF and SC) independently screened studies by title and abstract. Any disagreements were resolved through discussion and consensus. Full-text articles were subsequently assessed for eligibility, with discrepancies again addressed through consensus before data extraction. Additionally, the reference lists of included studies were reviewed to identify any further studies meeting the inclusion criteria.

### Data extraction

Data were extracted using a standardized data collection form that captured study-level characteristics, patient demographics, clinical presentation, imaging findings, additional diagnostic workup (including hormonal evaluation and cerebrospinal fluid analysis), systemic disease involvement, histopathological findings, treatment strategies, and clinical outcomes.

Data extraction was performed independently by two reviewers. One reviewer (JF) conducted manual data extraction using a standardized form. In parallel, a structured extraction support strategy was applied by a second reviewer (SC), using a predefined prompt in ChatGPT 5.5 (OpenAI, San Francisco, CA, USA) to assist in identifying and organizing study variables into a standardized spreadsheet. The prompts and extraction framework are described in the Supplementary Material. Agreement between both datasets was assessed and showed high concordance (95%; Cohen’s κ = 0.94). Any discrepant data were subsequently reviewed manually against the full-text articles and resolved by consensus.

### Critical appraisal

A formal risk-of-bias assessment was not performed because all included studies were case reports or case series, which are inherently high-risk.

### Data categorization and analysis

Variables were categorized as demographic, clinical, radiological, laboratory, systemic, or treatment-related, and coded as binary (presence/absence), categorical, or continuous as appropriate. For each variable, the denominator reflects the number of patients for whom the source report provided sufficient information to confirm presence or absence of that specific finding; patients whose source report did not address a given variable were excluded from its denominator rather than assumed negative. This approach was applied uniformly across demographic, clinical, radiological, and laboratory variables, and accounts for the varying denominators reported throughout Tables 1, 2, 3 and 4.

### Statistical analysis

Statistical analyses were conducted using RStudio (R Foundation for Statistical Computing, Vienna, Austria; 2024.09.1 + 394 (2024.09.1 + 394)). Categorical variables are reported as counts and percentages; continuous variables are reported as medians with interquartile ranges (IQR). Missing data was handled using complete case analysis. Missing data were not imputed, and percentages were calculated using the number of patients with available data for each variable as the denominator.

Two sensitivity analyses were performed to assess the robustness of the primary findings. First, the cohort was stratified by diagnostic certainty per the 2018 NCCG criteria, comparing patients meeting definite or probable criteria against those meeting only possible criteria; categorical variables were compared using Fisher’s exact test and continuous variables using the Mann-Whitney U test.

Second, the cohort was stratified into three publication eras selected to reflect two developments expected to most plausibly influence diagnostic and therapeutic practice: the increasing availability of MRI, for which the year 2000 was chosen as an approximate marker, and the publication of the current NCCG diagnostic criteria in 2018. This yielded three strata (1954–1999, 2000–2017, and 2018–2024) used to evaluate the influence of temporal heterogeneity on the study findings; categorical variables were compared using the chi-square test, with a Monte Carlo-simulated exact test substituted when expected cell counts were < 5, and continuous variables were compared using the Kruskal-Wallis test. A two-sided p-value < 0.05 was considered statistically significant. Full results of both sensitivity analyses are provided in Supplementary Tables 1 and 2.

## Results

### Study selection and demographics

**Fig. 1:**
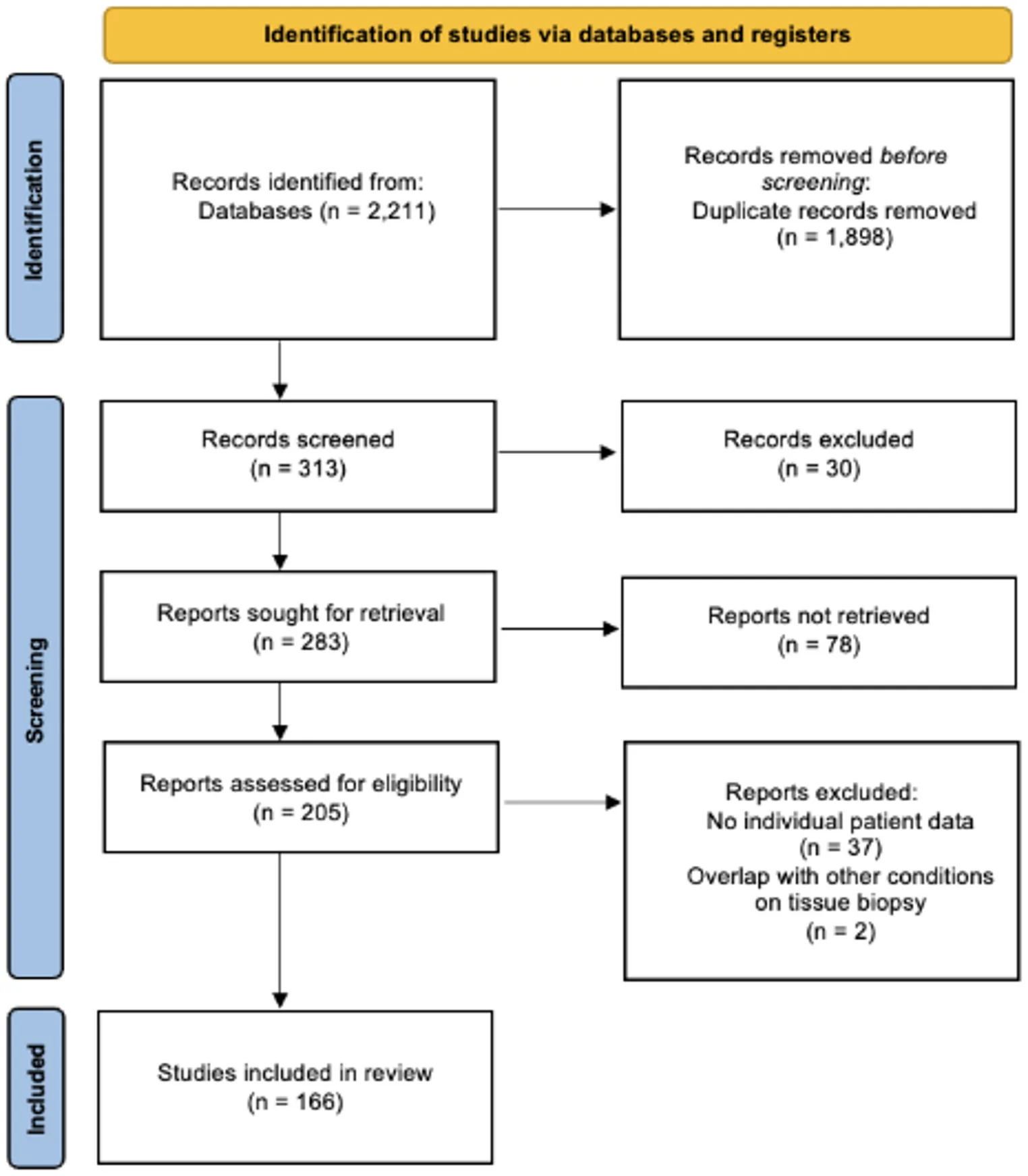
Flow diagram

The literature search yielded 2,211 records (PubMed: *n* = 399; Scopus: *n* = 808; Embase: *n* = 1,004). After removal of 1,898 duplicate records, 313 unique records remained and were screened by title and abstract, of which 30 were excluded. A total of 283 reports were sought for full-text retrieval; 78 were not retrieved. Of the 205 full-text articles assessed for eligibility, 39 were excluded due to lack of individual patient data (*n* = 37) or overlap with other conditions on histopathology (*n* = 2; one lymphoma and one neuroendocrine tumor). Ultimately, 166 studies were included in the qualitative synthesis. Figure 1 presents the flow diagram.

We included 274 patients across publications from 1954 to 2024. Of the 271 patients with documented sex, 145 were male (53.5%) and 126 female (46.5%). Median follow-up was 36 months (IQR 11–84). Most case reports originated from the United States (112; 40.9%), France (40; 14.6%), and Japan (34; 12.4%).

HP involvement was supported by neuroimaging (sellar mass, pituitary stalk thickening, or hypothalamic parenchymal involvement) in 187 patients (68.2%) and by endocrine/hormonal abnormality (anterior pituitary axis deficiency and/or AVP deficiency) in 229 (83.6%). Both radiologic and hormonal evidence were present in 164 patients (59.9%), with radiologic findings alone in 23 (8.4%) and hormonal abnormality alone in 65 (23.7%). Histopathological confirmation specific to the hypothalamic-pituitary region was obtained in 49 patients (17.9%).

### Diagnostic classification

The diagnostic classification of the cohort according to the 2018 NCCG criteria was: 72 patients (26.3%) met criteria for definite neurosarcoidosis, 131 (47.8%) for probable neurosarcoidosis, and 71 (25.9%) for possible neurosarcoidosis. The combined definite/probable cohort (203 patients, 74.1%), which comprises those with histopathological confirmation of sarcoidosis, either from central nervous system tissue or an extraneural site, formed the basis for the sensitivity analysis described below. Isolated neurosarcoidosis, defined as disease confined to the nervous system without evidence of systemic organ involvement, was identified in 37 patients (13.5%).

### Signs and symptoms

Table 1 summarizes the clinical presentation across the cohort. AVP deficiency (AVP-D) was the most frequently reported manifestation, identified in 147 of 274 patients (53.6%). Gonadal dysfunction (which includes decreased libido, erectile dysfunction, amenorrhea, galactorrhea, oligomenorrhea, early menopause, delayed puberty, failure of sexual development, and gynecomastia) was the most common anterior pituitary manifestation, identified in approximately 92 of 240 patients (38.3%). Headache was reported in 75 of 233 patients (32.2%), and fatigue in 71 of 233 (30.5%).

Among cranial nerve manifestations, visual loss (CN II) was the most frequent, identified in 87 of 241 patients (36.1%). Diplopia and/or strabismus (CN III, IV, or VI) occurred in 30 of 241 patients with available data (12.4%). Less common cranial nerve findings included vertigo and/or hearing loss (CN VIII; 18/240, 7.5%), facial palsy (CN VII; 12/240, 5.0%), facial numbness (CN V) and bulbar syndrome (CN IX–XII), each in 9 of 240 patients (3.8%), and olfactory dysfunction (CN I; 8/240, 3.3%). Cognitive impairment or altered mental status was present in 42 of 240 patients (17.5%), and motor weakness or focal deficits in 37 of 240 (15.4%). Autonomic/hypothalamic symptoms occurred in 21 of 240 patients (8.8%), and weight loss in 20 of 240 (8.3%). Seizures and nausea/vomiting were each reported in 19 of 240 patients (7.9%). Less frequent findings included psychiatric or behavioral symptoms (15/240, 6.2%), dizziness and anorexia (each 11/240, 4.6%), and weight gain (10/240, 4.2%).

**Table 1:** Demographic features and clinical presentation of hypophysitis in sarcoidosis

Characteristic	No. (%)
Demographic features (*n* = 274)	
Age, median (IQR), y	37 (28–48)
Sex (male)	145/271 (53.5%)
Signs and symptoms	
AVP-D	147/274 (53.6%)
Gonadal dysfunction†	92/240 (38.3%)
Visual loss (II CN)	87/241 (36.1%)
Headache	75/233 (32.2%)
Fatigue	71/233 (30.5%)
Cognitive impairment / altered mental status	42/240 (17.5%)
Weakness/motor deficit	37/240 (15.4%)
Diplopia and/or strabismus (III, IV, or VI CN)	30/241 (12.4%)
Autonomic/hypothalamic symptoms‡	21/240 (8.8%)
Weight loss	20/240 (8.3%)
Seizures	19/240 (7.9%)
Nausea/vomiting	19/240 (7.9%)
Vertigo and/or hearing loss (VIII CN)	18/240 (7.5%)
Psychiatric or behavioral symptoms	15/240 (6.2%)
Facial palsy (VII CN)	12/240 (5.0%)
Dizziness	11/240 (4.6%)
Anorexia	11/240 (4.6%)
Weight gain	10/240 (4.2%)
Facial numbness/pain (V CN)	9/240 (3.8%)
Bulbar syndrome (IX, X, XI, or XII CN)	9/240 (3.8%)
Olfactory dysfunction (I CN)	8/240 (3.3%)

† Includes decreased libido, erectile dysfunction, amenorrhea, galactorrhea, oligomenorrhea, early menopause, delayed puberty, failure of sexual development, and gynecomastia.

‡ Includes hypothermia/hyperthermia, hyperphagia, and hypersomnia.

For signs and symptoms extracted from free-text case descriptions (all rows except AVP-D, visual loss, headache, and fatigue), denominators reflect patients for whom the source report provided sufficient detail to confirm presence or absence of the finding, patients whose source report provided no such detail were excluded from the denominator.

CN cranial nerve.

### Systemic involvement

Systemic organ involvement was documented in 216 of 269 patients (80.3%) and is detailed in Table 2. The specific organ involvement was reported in 265 patients. The three most commonly affected organ systems were lymph nodes (136/265; 51.3%), lung (123/265; 46.4%), and skin (43/265; 16.2%). Ocular or orbital involvement (35/265; 13.2%) and sinonasal disease (27/265; 10.2%) were also frequently reported. Liver involvement was present in 21 of 265 patients (7.9%), bone or skeletal involvement in 13 (4.9%), and cardiac involvement in 10 (3.8%). Renal, splenic, salivary gland, gastrointestinal, and testicular involvement were each reported in fewer than 3% of cases.

**Table 2:** Systemic organ involvement in hypophysitis in sarcoidosis

Organ / System	No. (%)
Any systemic involvement (*n* = 269)	
Any extra-neurological organ involvement	216/269 (80.3%)
Specific organ involvement (*n* = 265)	
Lymph nodes (any)	136/265 (51.3%)
Lung / pulmonary	123/265 (46.4%)
Skin	43/265 (16.2%)
Eye / orbital	35/265 (13.2%)
Sinonasal	27/265 (10.2%)
Liver	21/265 (7.9%)
Bone / skeletal	13/265 (4.9%)
Cardiac	10/265 (3.8%)
Kidney / renal	7/265 (2.6%)
Spleen	7/265 (2.6%)
Salivary gland	5/265 (1.9%)
Gastrointestinal	5/265 (1.9%)
Testicular	4/265 (1.5%)

### Investigation findings

**Fig. 2:**
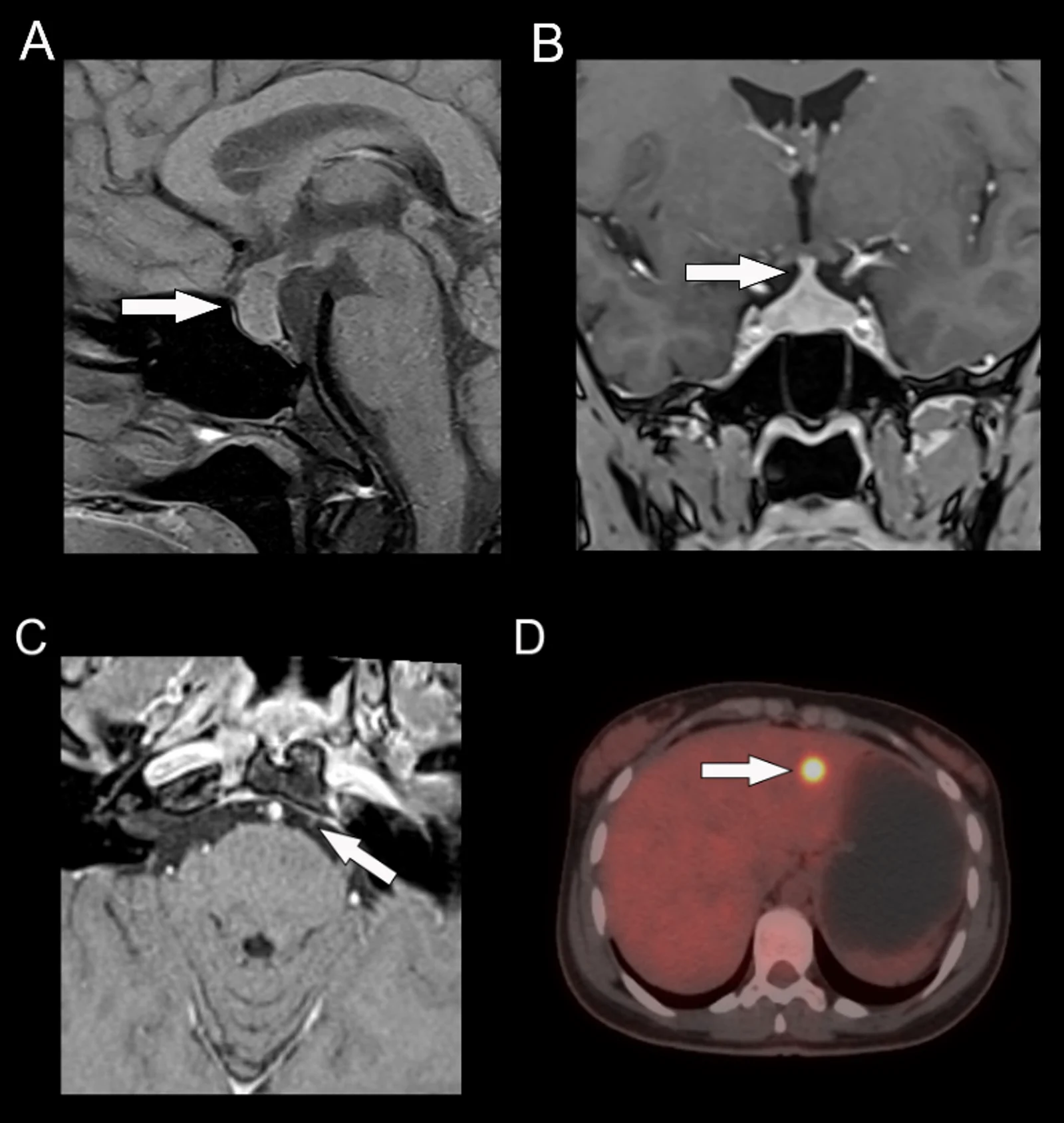
Characteristic imaging findings in a patient with hypophysitis due to neurosarcoidosis. Sagittal T1-weighted image (A) demonstrates a sellar lesion encompassing the pituitary gland (arrow), with absence of the normal T1 hyperintense signal of the posterior pituitary (neurohypophysis), consistent with loss of the posterior pituitary bright spot. Coronal postcontrast T1-weighted image (B) shows heterogeneous enhancement of the pituitary lesion (with a major axis of 20 mm), associated with pituitary stalk thickening (arrow). Axial postcontrast T1- weighted image (C) discloses abnormal enhancement of the distal cisternal portion of the left abducens nerve (arrow). FDG PET-CT image (D) reveals a hypermetabolic nodular lesion in the left hepatic lobe (arrow).

Available data on investigation findings are summarized in Table 3. MRI data were retrieved in 209 of 261 patients (80.1%). Among those with MRI findings reported, pituitary stalk thickening was the most common finding, present in 84 of 195 patients (43.1%). A sellar or suprasellar mass was identified in 71 of 195 patients (36.4%), with a lesion exceeding 10 mm in 33 of 147 cases (22.4%). Figure 2 illustrates common imaging findings. 

At least one anterior pituitary axis was affected in 193 of 274 patients (70.4%). FSH/LH deficiency (central hypogonadism) was the most frequent endocrine abnormality, identified in 74.1% (149/201), followed by TSH deficiency in 63.6% (131/206), hyperprolactinemia in 58.1% (115/198), ACTH deficiency in 52.8% (102/193), and GH deficiency in 46.1% (88/191). Three or more axes were affected in 117 patients (42.7%), including the 39 (14.2%) with panhypopituitarism (deficiency of all five pituitary axes).

Hypercalcemia was noted in 8 patients (2.9%).CSF analysis was available in a subset of patients, and elevated protein was the most common abnormality, identified in 51 of 74 patients (68.9%), with a median protein level of 101 mg/dL (IQR 76-224). We only had two reported results for sIL2r in CSF, one with elevated levels and another within the normal range. 

**Table 3:** Investigation findings of hypophysitis in sarcoidosis

Characteristic	No. of measures available	No. of patients (%)
Cerebrospinal fluid characteristics		
Elevated protein (> 40 mg/dL)	74	51 (68.9%)
Protein, median (IQR), mg/dL	41	101 (76–224)
Pleocytosis (WBC > 5 cells/mm³)	74	44 (59.5%)
WBC count, median (IQR), cells/mm³	36	21.5 (8.8–60.5)
Low glucose	68	12 (17.6%)
CSF ACE elevated	25	11 (44.0%)
Oligoclonal bands†	74	8 (10.8%)
Serum laboratory findings		
ACE elevated	75	35 (46.7%)
* ACE*,* median (IQR)*,* U/L*	42	58.5(31.4–114.8)
IL-2R elevated	14	10 (71.4%)
* IL-2R*,* median (IQR)*,* U/L*	14	1130 (538–1946)
ESR, median (IQR), mm/h	29	40 (30–52)
CRP, median (IQR), mg/L	11	17 (2–30)
Hypercalcemia	274	8 (2.9%)
Endocrine findings‡		
FSH/LH deficiency (central hypogonadism)	201	149 (74.1%)
TSH deficiency (central hypothyroidism)	206	131 (63.6%)
Hyperprolactinemia	198	115 (58.1%)
ACTH deficiency (central hypocortisolism)	193	102 (52.8%)
GH deficiency	191	88 (46.1%)
Panhypopituitarism (all 5 axes affected)	274	39 (14.2%)
Radiological findings		
MRI performed	261	209 (80.1%)
Pituitary stalk thickening	195	84 (43.1%)
Sellar or suprasellar mass	195	71 (36.4%)
* Mass > 10 mm*	147	33 (22.4%)
Biopsy		
Biopsy performed	274	203 (74.1%)
Biopsy sites¶		
Lymph node (any)	203	73 (36.0%)
Pituitary/ sellar / suprasellar	203	45 (22.2%)
Lung / bronchial/ transbronchial	203	41 (20.2%)
Brain/ hypothalamus / meningeal	203	25 (12.3%)
Skin	203	18 (8.9%)
Sinonasal	203	17 (8.4%)
Liver	203	8 (3.9%)
Other	203	13 (6.4%)

ACE angiotensin-converting enzyme, ACTH adrenocorticotropic hormone, CRP C-reactive protein, CSF cerebrospinal fluid, ESR erythrocyte sedimentation rate, FSH follicle-stimulating hormone, GH growth hormone, IQR interquartile range, IL-2R interleukin-2 receptor, LH luteinizing hormone, MRI magnetic resonance imaging, TSH thyroid-stimulating hormone, WBC white blood cell.

† Oligoclonal bands reported in a subset of patients who underwent lumbar puncture, positivity/negativity not consistently specified in source reports.

‡ Endocrine denominators vary due to incomplete hormonal workup across case reports. Deficiency is defined as below the lower limit of normal or clinically reported as deficient.

¶ Biopsy site data are available for 203 patients who underwent biopsy, categories are not mutually exclusive, as some patients had biopsies from more than one site.

## Treatment and prognosis

Follow-up data duration was available for 156 of 274 patients (56.9%), with a median follow-up of 36 months (IQR 11–84). Disease course (monophasic vs. recurrent) was documented in 216 patients, among which 123 (56.9%) had a monophasic course, while 93 (43.1%) experienced at least one recurrence. Recurrences were predominantly neurological (88/93, 94.6%), with non-neurological recurrence reported in only 5 patients (5.4%). The median time to recurrence was 12 months (IQR 2–28) (Table 4).

Corticosteroids were the cornerstone of acute treatment, administered in 208 of 220 patients with available data (94.5%). Desmopressin was prescribed acutely in 76 of 220 patients (34.5%), and acute hormone replacement therapy was initiated in 59 of 220 patients (26.8%). For maintenance therapy, corticosteroids remained the most used agent, prescribed in 149 of 174 patients (85.6%), with prednisone and prednisolone being the most frequently reported formulations. Methotrexate was the most common steroid-sparing immunosuppressant, used in 39 of 174 patients (22.4%), followed by infliximab in 18 (10.3%), mycophenolate mofetil in 17 (9.8%), azathioprine in 15 (8.6%), cyclophosphamide in 6 (3.4%), and cyclosporine in 5 (2.9%) (Table 4). Twenty-four patients (13.8%) received more than one steroid-sparing agent, most commonly methotrexate combined with infliximab (n=4).

Chronic hormonal replacement was common and required in 63 of 174 patients (36.2%). Among patients with available data on therapy type, thyroid supplementation was the most frequent (55 patients), followed by sex hormone replacement (31 patients); these categories were not mutually exclusive, consistent with the frequent co-occurrence of multiple anterior pituitary axis deficiencies in this population. Chronic desmopressin use was defined as the long-term prescription of any vasopressin analogs, including desmopressin, pitressin, and lypressin, and was recorded in 53 of 174 patients (30.5%) (Table 4). 

In comparative analysis, recurrence occurred in 75% of infliximab-treated patients (12/16 with available follow-up) versus 47.3% of those receiving other immunosuppressants (61/129; p=0.061), with no significant difference in chronic hormonal replacement (27.8% vs. 36.4%; p=0.604) or desmopressin dependence (22.2% vs 24.6%; p=1.000). 88.9% of infliximab-treated patients had received another immunosuppressant concurrently or previously.

**Table 4:** Recurrence, chronic hormonal dependence, and maintenance therapy in hypophysitis in sarcoidosis

Characteristic	No. (%)
Recurrence (*n* = 216)	
Overall recurrence	93/216 (43.1%)
Recurrence type — neurological	88/93 (94.6%)
Recurrence type — non-neurological	5/93 (5.4%)
Chronic Hormonal Dependence (*n* = 174)	
Hormonal replacement therapy	63/174 (36.2%)
Desmopressin*	53/174 (30.5%)
Maintenance Therapy (*n* = 174)	
Corticosteroids	149/174 (85.6%)
Methotrexate	39/174 (22.4%)
Infliximab	18/174 (10.3%)
Mycophenolate mofetil	17/174 (9.8%)
Azathioprine	15/174 (8.6%)
Cyclophosphamide	6/174 (3.4%)
Cyclosporine	5/174 (2.9%)
Radiotherapy	4/174 (2.3%)
Rituximab	1/174 (0.6%)

* Includes the use of desmopressin, vasopressin, and pitressin

## Sensitivity analyses

Two sensitivity analyses were performed to assess the robustness of the primary findings First, patients meeting definite or probable neurosarcoidosis criteria (n=203) were compared against those meeting only possible criteria (n=71). The core clinical and endocrine phenotype was preserved between groups: recurrence (42.5% vs. 44.9%, p=0.87), all five individual anterior pituitary hormone axes, panhypopituitarism, and systemic organ involvement did not differ significantly. Differences that did reach significance were sellar/suprasellar mass, lesion size >10 mm, headache, visual loss, and fatigue, which were all more frequent among biopsy-confirmed patients. These results are available in Supplementary Table 1. 

Second, the cohort was stratified into three publication eras (1954–1999, n=83; 2000–2017, n=118; 2018–2024, n=73). MRI utilization increased markedly across eras (39.7% vs. 94.9% vs. 97.1%, p<0.001), and methotrexate (0% vs. 30.1% vs. 27.5%, p<0.001) and infliximab (0% vs. 11.8% vs. 17.5%, p=0.028) were used exclusively in patients reported after 2000. Median age at diagnosis (31 vs. 38 vs. 44 years, p<0.001) and AVP-D detection (38.6% vs. 56.8% vs. 65.8%, p=0.002) also increased over time. Recurrence appeared lower in the most recent era (52.5% vs. 47.4% vs. 26.7%, p=0.009), but this coincided with a shorter median follow-up duration in the same period (49 vs. 36 vs. 9 months, p=0.003). The five individual anterior pituitary hormone axes, panhypopituitarism, pituitary stalk thickening, sellar/suprasellar mass, and systemic organ involvement did not differ significantly across eras. Results are available in Supplementary Table 2. 

## Discussion

In this systematic review, including 274 patients with sarcoidosis-related hypophysitis from 166 studies, AVP deficiency was the most frequent clinical manifestation, followed by visual symptoms, headache, fatigue, and anterior pituitary hormone deficiencies, particularly central hypogonadism. MRI most demonstrated pituitary stalk thickening and sellar/suprasellar masses, while CSF analysis frequently showed elevated protein and pleocytosis. Systemic involvement was identified in most patients, predominantly affecting lymph nodes and lungs. Corticosteroids were the mainstay of treatment; however, recurrence was observed in nearly half of patients with available follow-up, and long-term hormonal replacement and desmopressin dependence were common, highlighting the chronic morbidity associated with the disease.

The observed predominance of AVP-D in our study is consistent with early reports [13, 14]. More recent series and multicenter registries [3, 8–10] have begun to challenge this paradigm, suggesting that gonadal axis dysfunction, particularly secondary hypogonadism, may be at least as common and potentially more prevalent when systematically sought. This difference could be explained by the possible underreporting of gonadal dysfunction symptoms in older reports. Complaints such as erectile dysfunction, decreased libido, and menstrual irregularities are often not volunteered by patients and are frequently absent from case reports unless the authors included a targeted endocrine history. Our data, with gonadal dysfunction recorded in 38.3% of patients, likely underestimates the true prevalence of this manifestation, and the paradigm shift observed in more recent literature is probably more a methodological advance than an epidemiological one. Therefore, routine, structured assessment of all anterior pituitary axes should be considered at presentation and follow-up in patients with hypothalamic-pituitary sarcoidosis.

Laboratory and radiological investigations in our cohort were highly heterogeneous, reflecting the absence of a standardized diagnostic algorithm for sarcoidosis-related hypophysitis. Our findings also highlight the limited sensitivity of currently available fluid biomarkers in this setting. Elevated serum ACE levels were identified in only 46% of patients, whereas CSF ACE elevation was observed in 44%, consistent with current neurosarcoidosis guidelines that emphasize the limited diagnostic utility of ACE measurements. These findings reinforce the importance of histopathological confirmation whenever feasible, which is often possible from extracranial sites given the systemic nature of sarcoidosis. Accordingly, a comprehensive systemic evaluation with computed tomography and, when available, FDG-PET imaging should be considered in patients presenting with hypophysitis potentially related to sarcoidosis, both to identify occult systemic involvement and to guide safer, more accessible biopsy targets [15].

Corticosteroids were the cornerstone of acute management across the cohort, consistent with current guidelines and expert consensus. Among steroid-sparing and second-line agents, methotrexate, azathioprine, and mycophenolate mofetil were the most frequently employed. Notably, infliximab was used in only 18 patients of our cohort (10.3%). This low rate of anti-TNF use likely reflects older publication dates of included reports, as evidence for infliximab use in neurosarcoidosis has consolidated over the past decade [16, 17]. Most included patients used infliximab as a rescue agent in a severe or relapsing disease population rather than as a first-line choice, which might introduce selection bias into this review and obscure the potential benefit of infliximab in patients with sarcoidosis and hypophysitis. More studies are required to determine whether early use of anti-TNF agents reduces the frequency of irreversible endocrine deficits in these patients.

This systematic review has several limitations. First, the available evidence consists predominantly of case reports and small retrospective series, which are inherently susceptible to publication bias and may preferentially report patients with favorable outcomes or unusual presentations. Notably, our cohort did not include any patients with asymptomatic radiological manifestations of the disease, likely reflecting this same publication bias. Second, our cohort included patients across the full spectrum of diagnostic certainty defined by the 2018 NCCG criteria, including those classified as only “possible” neurosarcoidosis. We assessed whether this affected our estimates through a sensitivity analysis restricting the cohort to definite/probable cases, described below. Third, substantial heterogeneity was observed across included studies in endocrine assessment. Hormonal evaluation was frequently incomplete or inconsistently reported, likely leading to underestimation of endocrine dysfunction. Nevertheless, despite this limitation, a large proportion of patients ultimately required long-term hormonal replacement therapy, underscoring the clinical relevance of pituitary dysfunction in this population.

Similarly, treatment approaches varied considerably across reports, including differences in corticosteroid dosing, tapering regimens, treatment duration, and indications for escalation to steroid-sparing immunosuppressive agents, limiting meaningful comparisons of therapeutic effectiveness. This is particularly relevant to the comparative infliximab findings reported: infliximab was predominantly used as salvage therapy in patients with refractory and recurrent disease, and nearly 90% of infliximab-treated patients had received another immunosuppressant concurrently or previously. The higher recurrence rate observed in this group therefore most likely reflects greater baseline disease severity and treatment-refractoriness among patients selected for infliximab rather than reduced drug efficacy. Given this confounding by indication and the observational, non-randomized nature of the underlying data, direct comparisons of treatment efficacy between therapeutic groups cannot be made, and the reported percentages should be interpreted descriptively rather than as evidence of comparative effectiveness.

Additionally, follow-up duration was inconsistently reported and highly variable across studies (median 36 months, IQR 11–84), potentially leading to an underestimation of recurrence rates, particularly given the recognized occurrence of delayed relapses during corticosteroid tapering in neurosarcoidosis. However, despite this limitation, relapses remained frequent, occurring in more than 40% of patients. Lastly, our cohort spans publications from 1954 to 2024, a nearly 70-year period marked by substantial advances in neuroimaging, endocrine testing, and treatment for neurosarcoidosis. We addressed this temporal heterogeneity through a second sensitivity analysis stratifying the cohort by publication era, also described below.

The robustness of our findings was further supported by two sensitivity analyses. Restricting the cohort to patients meeting definite or probable neurosarcoidosis criteria did not materially change the frequency of endocrine dysfunction, systemic involvement, or recurrence, addressing concerns that inclusion of possible cases might bias our estimates. Sellar/suprasellar mass, lesion size > 10 mm, headache, and visual loss were all significantly more frequent among biopsy-confirmed patients, suggesting that more prominent lesions both produced more mass-effect-related symptoms and were more likely to prompt biopsy or resection. Fatigue was also more frequent among biopsy-confirmed patients; however, as a nonspecific systemic symptom without a direct anatomic link to lesion size, this likely reflects a greater overall symptomatic burden in this group rather than the same mass-effect mechanism, or may represent a chance finding given the number of comparisons performed.

Stratifying the cohort by publication era, MRI utilization and steroid-sparing/biologic therapy use increased substantially in more recent decades. Importantly, the core clinical and endocrine phenotype remained stable across eras, supporting the generalizability of our pooled estimates. Several other era-associated differences warrant caution in interpretation: the apparent decline in recurrence in the most recent era is confounded by a correspondingly shorter follow-up duration in report and should not be read as sole evidence of improved long-term outcomes.

This study also has important strengths. A major strength is the exclusive use of individual patient-level data rather than aggregated study-level outcomes, allowing a more detailed characterization of clinical manifestations, endocrine dysfunction, imaging findings, and treatment outcomes, while also reducing the risk of duplicate case inclusion across publications. In addition, this represents the largest study to date specifically focused on sarcoidosis-related hypophysitis. Our findings further highlight the likely underrecognized burden of gonadal dysfunction in these patients and support the need for future studies evaluating steroid-sparing therapies, particularly anti-TNF agents, in refractory or relapsing disease. Overall, this study provides a comprehensive synthesis of the main clinical features, investigation findings, and treatment outcomes of sarcoidosis-related hypophysitis, offering practical information to support clinicians managing patients with hypophysitis.

## Supplementary Information

Below is the link to the electronic supplementary material.

Supplementary Material 1

## Data Availability

No datasets were generated or analysed during the current study.
