# Supplementary material for: Clinical presentation, investigation findings, and outcomes of hypophysitis in sarcoidosis: a systematic review
Source: Pituitary. 2026 Aug 20;29(5):148. doi: 10.1007/s11102-026-01753-y (PMC13493403; doi:10.1007/s11102-026-01753-y)
Supplement: Supplementary file 1 [file 11102_2026_1753_MOESM1_ESM.pdf]

Supplementary Material

1. Search strategies

Three electronic databases were searched for studies on hypophysitis in neurosarcoidosis (PubMed/MEDLINE, Embase, and Scopus). The search did not have a start date limit ("from inception"" and was last updated on 20th January 2025. The following search terms were used in each database: ("sarcoidosis" OR "neurosarcoidosis" OR "neurologic sarcoidosis") AND ("hypophysis" OR "pituitary" OR "pituitary inflammation" OR "pituitary disease" OR "hypophysitis" OR "granulomatous hypophysitis" OR "pituitary dysfunction" OR "hypopituitarism" OR "panhypopituitarism").

2. Structured Data Extraction Prompt ChatGPT Version 5.5

A customized GPT-based extraction framework was developed to standardize data collection from eligible articles included in this systematic review. The model was instructed to extract predefined demographic, clinical, laboratory, imaging, treatment, and follow-up variables from each publication and output the results in a structured format suitable for spreadsheet analysis.

The following variables were extracted for each case report or study participant:

Article and demographic data:

- n_article: article identification number
- year: year of publication (YYYY format)
- country: country of origin (full English name)
- age: age in years (integer)
- sex: coded as 0 = female, 1 = male

Clinical manifestations:

- headache: 0 = no, 1 = yes
- visual_loss: 0 = no, 1 = yes
- diplopia: 0 = no, 1 = yes
- diabetes_insipidus: 0 = no, 1 = yes
- fatigue: 0 = no, 1 = yes
- other: description of additional neurological manifestations

Imaging findings:

- mri: 0 = no magnetic resonance imaging performed, 1 = MRI performed
- mri_result: textual description of MRI findings
- sellar_mass: 0 = no, 1 = yes
- more_10_mm: 1 if lesion size >10 mm, 0 if ≤10 mm
- stalk_thickening: 0 = no, 1 = yes

Pituitary hormonal assessment:

- gh, tsh, acth, fsh_lh, prolactine: coded as 0 = normal, 1 = altered

Laboratory findings:

- ace: serum angiotensin-converting enzyme status (0 = normal, 1 = altered)
- ace_value: exact serum ACE value when reported
- il_2r: serum interleukin-2 receptor status (0 = normal, 1 = altered)
- il_2r_value: exact serum value when available
- crp: C-reactive protein value
- esr: erythrocyte sedimentation rate value

Cerebrospinal fluid (CSF) analysis:

- csf_cells: cell count result
- csf_cells_type: 0 = polymorphonuclear/neutrophilic, 1 = lymphocytic predominance
- csf_cells_value: exact laboratory value
- csf_protein: protein status (0 = normal, 1 = altered)
- csf_protein_value: exact protein value
- csf_glucose: glucose status (0 = normal, 1 = altered)
- csf_il_2r: interleukin-2 receptor result
- csf_il_2r_value: exact value
- csf_ace: CSF ACE status (0 = normal, 1 = altered)

Neurological and systemic involvement:

- other_neuro: 0 = no, 1 = yes
- other_neuro_desc: description of additional neurosarcoidosis manifestations
- systemic: 0 = no, 1 = yes
- other_systemic: 0 = no, 1 = yes
- other_systemic_desc: description of additional systemic manifestations
- biopsy_site: biopsy site

Outcomes and treatment:

- recurrence: 0 = no, 1 = yes
- follow_up: follow-up duration in months
- time_to_recurrence: time to recurrence in months
- acute_treatment: acute treatment administered (full drug name)
- chronic_treatment: chronic treatment administered (full drug name)

Standardized extraction rules:

- To ensure consistency across studies, the following extraction rules were applied:
- Missing or unavailable information was coded as "-".
- Hormonal variables were binarized as 0 = normal and 1 = altered, regardless of numerical laboratory values.
- MRI findings were described textually in the mri_result field.
- Lesions larger than 10 mm were coded as more_10_mm = 1; lesions ≤10 mm were coded as 0.
- CSF cellular predominance was classified as 0 for polymorphonuclear/neutrophilic and 1 for lymphocytic patterns.
- Exact laboratory values were transcribed whenever available, preserving reported numerical precision (one decimal place when applicable).
- Data were extracted and exported in a semicolon-separated format compatible with spreadsheet software for downstream quantitative analysis.

3. Supplementary Table. Sensitivity Analysis: Full Cohort vs. Definite/Probable (Retained) vs. Possible (Excluded) Neurosarcoidosis

Bold rows indicate p<0.05 (Fisher's exact test for categorical variables; Mann-Whitney U for continuous variables), comparing Retained vs. Excluded.

Variable	Full cohort (N=274)	Retained: Def+Prob (n=203)	Excluded: Possible (n=71)	p
**Demographics**				
Age (y)	37.0 (28.0-48.0), n=273	36.0 (27.0-48.0), n=202	39.0 (32.5-48.0), n=71	0.078
**Sex (male)**	**145/271 (53.5%)**	**117/203 (57.6%)**	**28/68 (41.2%)**	**0.024**
**Symptoms**				
AVP-D	147/274 (53.6%)	115/203 (56.7%)	32/71 (45.1%)	0.099
**Visual loss**	**87/241 (36.1%)**	**72/174 (41.4%)**	**15/67 (22.4%)**	**0.007**
**Headache**	**75/233 (32.2%)**	**62/167 (37.1%)**	**13/66 (19.7%)**	**0.012**
**Fatigue**	**71/233 (30.5%)**	**60/167 (35.9%)**	**11/66 (16.7%)**	**0.004**
Gonadal dysfunction	92/240 (38.3%)	71/173 (41.0%)	21/67 (31.3%)	0.185
Cognitive/altered MS	42/240 (17.5%)	29/173 (16.8%)	13/67 (19.4%)	0.705
Weakness/motor deficit	37/240 (15.4%)	24/173 (13.9%)	13/67 (19.4%)	0.320
Weight loss	20/240 (8.3%)	18/173 (10.4%)	2/67 (3.0%)	0.071
Weight gain	10/240 (4.2%)	9/173 (5.2%)	1/67 (1.5%)	0.291
Psychiatric/behavioral	15/240 (6.2%)	13/173 (7.5%)	2/67 (3.0%)	0.246
Seizures	19/240 (7.9%)	7/173 (4.0%)	12/67 (17.9%)	<0.001
**Nausea/vomiting**	**19/240 (7.9%)**	**18/173 (10.4%)**	**1/67 (1.5%)**	**0.029**
Autonomic/hypothalamic	21/240 (8.8%)	15/173 (8.7%)	6/67 (9.0%)	1.000

Vertigo/hearing loss (CN VIII)	18/240 (7.5%)	12/173 (6.9%)	6/67 (9.0%)	0.591
**Facial numbness (CN V)**	**9/240 (3.8%)**	**3/173 (1.7%)**	**6/67 (9.0%)**	**0.016**
Dizziness	11/240 (4.6%)	9/173 (5.2%)	2/67 (3.0%)	0.732
Anorexia	11/240 (4.6%)	9/173 (5.2%)	2/67 (3.0%)	0.732
Bulbar syndrome	9/240 (3.8%)	6/173 (3.5%)	3/67 (4.5%)	0.712
Facial palsy (CN VII)	12/240 (5.0%)	8/173 (4.6%)	4/67 (6.0%)	0.743
**Olfactory dysfunction (CN I)**	**8/240 (3.3%)**	**3/173 (1.7%)**	**5/67 (7.5%)**	**0.041**
Diplopia/strabismus (sum of H + AA)	30/241 (12.4%)	22/174 (12.6%)	8/67 (11.9%)	1.000
**Systemic**				
Any systemic involvement	216/269 (80.3%)	166/203 (81.8%)	50/66 (75.8%)	0.290
Lymph nodes	136/265 (51.3%)	105/199 (52.8%)	31/66 (47.0%)	0.478
Lung	123/265 (46.4%)	89/199 (44.7%)	34/66 (51.5%)	0.393
Skin	43/265 (16.2%)	31/199 (15.6%)	12/66 (18.2%)	0.700
Liver	21/265 (7.9%)	19/199 (9.5%)	2/66 (3.0%)	0.115
Spleen	7/265 (2.6%)	7/199 (3.5%)	0/66 (0.0%)	0.198
Eye/orbital	35/274 (12.8%)	26/203 (12.8%)	9/71 (12.7%)	1.000
Sinonasal	27/274 (9.9%)	21/203 (10.3%)	6/71 (8.5%)	0.818
Bone/skeletal	13/274 (4.7%)	11/203 (5.4%)	2/71 (2.8%)	0.525
Cardiac	10/274 (3.6%)	7/203 (3.4%)	3/71 (4.2%)	0.722
Kidney/renal	7/274 (2.6%)	6/203 (3.0%)	1/71 (1.4%)	0.681
Salivary gland	6/274 (2.2%)	6/203 (3.0%)	0/71 (0.0%)	0.344
Gastrointestinal	5/274 (1.8%)	5/203 (2.5%)	0/71 (0.0%)	0.332
Testicular	4/274 (1.5%)	4/203 (2.0%)	0/71 (0.0%)	0.575
**Imaging**				
**MRI performed**	**209/261 (80.1%)**	**153/200 (76.5%)**	**56/61 (91.8%)**	**0.010**
Stalk thickening	84/195 (43.1%)	68/153 (44.4%)	16/42 (38.1%)	0.487
**Sellar/suprasellar mass**	**71/195 (36.4%)**	**62/153 (40.5%)**	**9/42 (21.4%)**	**0.029**

**Mass >10mm**	**33/147 (22.4%)**	**30/111 (27.0%)**	**3/36 (8.3%)**	**0.021**
**Endocrine**				
GH deficiency	88/191 (46.1%)	75/154 (48.7%)	13/37 (35.1%)	0.147
TSH deficiency	131/206 (63.6%)	106/166 (63.9%)	25/40 (62.5%)	0.857
ACTH deficiency	102/193 (52.8%)	85/157 (54.1%)	17/36 (47.2%)	0.466
FSH/LH deficiency	149/201 (74.1%)	124/165 (75.2%)	25/36 (69.4%)	0.530
Hyperprolactinemia	115/198 (58.1%)	91/161 (56.5%)	24/37 (64.9%)	0.460
Panhypopituitarism (all 5 axes)	39/274 (14.2%)	32/203 (15.8%)	7/71 (9.9%)	0.244
**CSF**				
Pleocytosis	44/74 (59.5%)	39/67 (58.2%)	5/7 (71.4%)	0.694
**Elevated CSF protein**	**51/74 (68.9%)**	**50/67 (74.6%)**	**1/7 (14.3%)**	**0.003**
Low CSF glucose	12/68 (17.6%)	12/62 (19.4%)	0/6 (0.0%)	0.581
CSF ACE elevated	11/25 (44.0%)	8/20 (40.0%)	3/5 (60.0%)	0.623
Oligoclonal bands	8/74 (10.8%)	7/67 (10.4%)	1/7 (14.3%)	0.567
**Serum markers**				
Serum ACE elevated	35/75 (46.7%)	27/61 (44.3%)	8/14 (57.1%)	0.554
IL-2R elevated	10/14 (71.4%)	7/9 (77.8%)	3/5 (60.0%)	0.580
Hypercalcemia	9/274 (3.3%)	8/203 (3.9%)	1/71 (1.4%)	0.454
**Continuous labs**				
CSF WBC count	21.5 (8.8-60.5), n=36	21.0 (8.0-65.0), n=33	50.0 (35.0-54.5), n=3	0.423
CSF protein	101.0 (76.0-224.0), n=41	101.0 (75.8-218.8), n=40	757.0 (757.0-757.0), n=1	0.139
Serum ACE value	58.5 (31.4-114.8), n=42	55.0 (31.7-110.5), n=31	67.0 (27.4-111.5), n=11	1.000
IL-2R value	1130.0 (538.2-1945.5), n=14	1108.0 (575.0-2124.0), n=9	1152.0 (500.0-1250.0), n=5	0.699
ESR	40.0 (30.0-52.0), n=29	38.0 (30.0-52.0), n=25	46.0 (37.8-52.0), n=4	0.752
CRP	17.1 (2.0-29.2), n=12	4.0 (2.0-17.3), n=9	29.0 (25.5-89.5), n=3	0.096
**Recurrence**				
Overall recurrence	93/216 (43.1%)	71/167 (42.5%)	22/49 (44.9%)	0.870

Recurrence type - neurological	88/93 (94.6%)	67/71 (94.4%)	21/22 (95.5%)	1.000
**Maintenance Therapy**				
Hormonal replacement therapy	63/174 (36.2%)	52/144 (36.1%)	11/30 (36.7%)	1.000
Desmopressin	53/174 (30.5%)	40/144 (27.8%)	13/30 (43.3%)	0.126
**Corticosteroids**	**149/168 (88.7%)**	**126/138 (91.3%)**	**23/30 (76.7%)**	**0.049**
Methotrexate	39/172 (22.7%)	32/142 (22.5%)	7/30 (23.3%)	1.000
Infliximab	18/174 (10.3%)	17/144 (11.8%)	1/30 (3.3%)	0.319
Mycophenolate mofetil	17/174 (9.8%)	13/144 (9.0%)	4/30 (13.3%)	0.499
Azathioprine	15/174 (8.6%)	15/144 (10.4%)	0/30 (0.0%)	0.077
Cyclophosphamide	6/174 (3.4%)	6/144 (4.2%)	0/30 (0.0%)	0.592
Cyclosporine	5/174 (2.9%)	4/144 (2.8%)	1/30 (3.3%)	1.000
Radiotherapy	4/174 (2.3%)	4/144 (2.8%)	0/30 (0.0%)	1.000
Rituximab	1/174 (0.6%)	1/144 (0.7%)	0/30 (0.0%)	1.000

4. Supplementary Table. Sensitivity Analysis: Temporal Heterogeneity Across Three Publication Eras

Bold rows indicate p<0.05 (chi-square test, or Monte Carlo simulated p-value when expected cell counts were <5, for categorical variables; Kruskal-Wallis test for continuous variables), across the three publication-era strata.

Variable	1954-1999	2000-2017	2018-2024	p
**Demographics**				
Age (y)	31.0 (26.5-38.0), n=83	38.0 (31.0-48.0), n=117	44.0 (32.0-55.0), n=73	<0.001
Sex (male)	36/83 (43.4%)	66/115 (57.4%)	43/73 (58.9%)	0.083
**Symptoms**				
**AVP-D**	**32/83 (38.6%)**	**67/118 (56.8%)**	**48/73 (65.8%)**	**0.002**
Visual loss	37/83 (44.6%)	26/85 (30.6%)	24/73 (32.9%)	0.133
Headache	27/75 (36.0%)	27/85 (31.8%)	21/73 (28.8%)	0.638
Fatigue	22/75 (29.3%)	28/85 (32.9%)	21/73 (28.8%)	0.823

**Gonadal dysfunction**	**45/83 (54.2%)**	**28/84 (33.3%)**	**19/73 (26.0%)**	**<0.001**
Cognitive/altered MS	10/83 (12.0%)	18/84 (21.4%)	14/73 (19.2%)	0.253
Weakness/motor deficit	13/83 (15.7%)	12/84 (14.3%)	12/73 (16.4%)	0.930
Weight loss	10/83 (12.0%)	3/84 (3.6%)	7/73 (9.6%)	0.126
Weight gain	5/83 (6.0%)	3/84 (3.6%)	2/73 (2.7%)	0.570
Psychiatric/behavioral	6/83 (7.2%)	7/84 (8.3%)	2/73 (2.7%)	0.376
Seizures	10/83 (12.0%)	5/84 (6.0%)	4/73 (5.5%)	0.225
Nausea/vomiting	10/83 (12.0%)	5/84 (6.0%)	4/73 (5.5%)	0.225
Autonomic/hypothalamic	7/83 (8.4%)	10/84 (11.9%)	4/73 (5.5%)	0.361
Vertigo/hearing loss (CN VIII)	9/83 (10.8%)	8/84 (9.5%)	1/73 (1.4%)	0.055
Facial numbness (CN V)	6/83 (7.2%)	2/84 (2.4%)	1/73 (1.4%)	0.126
Dizziness	4/83 (4.8%)	1/84 (1.2%)	6/73 (8.2%)	0.110
Anorexia	6/83 (7.2%)	1/84 (1.2%)	4/73 (5.5%)	0.157
Bulbar syndrome	5/83 (6.0%)	2/84 (2.4%)	2/73 (2.7%)	0.440
Facial palsy (CN VII)	6/83 (7.2%)	4/84 (4.8%)	2/73 (2.7%)	0.460
Olfactory dysfunction (CN I)	8/83 (9.6%)	0/84 (0.0%)	0/73 (0.0%)	<0.001
Diplopia/strabismus (sum of H + AA)	12/83 (14.5%)	12/85 (14.1%)	6/73 (8.2%)	0.423
**Systemic**				
Any systemic involvement	71/82 (86.6%)	89/114 (78.1%)	56/73 (76.7%)	0.223
Lymph nodes	46/78 (59.0%)	52/114 (45.6%)	38/73 (52.1%)	0.189
Lung	39/78 (50.0%)	51/114 (44.7%)	33/73 (45.2%)	0.750
Skin	19/78 (24.4%)	15/114 (13.2%)	9/73 (12.3%)	0.067
Liver	10/78 (12.8%)	7/114 (6.1%)	4/73 (5.5%)	0.161
Spleen	1/78 (1.3%)	2/114 (1.8%)	4/73 (5.5%)	0.247
**Eye/orbital**	**13/83 (15.7%)**	**19/118 (16.1%)**	**3/73 (4.1%)**	**0.035**
Sinonasal	5/83 (6.0%)	21/118 (17.8%)	1/73 (1.4%)	<0.001
Bone/skeletal	4/83 (4.8%)	4/118 (3.4%)	5/73 (6.8%)	0.550

Cardiac	2/83 (2.4%)	6/118 (5.1%)	2/73 (2.7%)	0.560
Kidney/renal	2/83 (2.4%)	3/118 (2.5%)	2/73 (2.7%)	1.000
Salivary gland	2/83 (2.4%)	2/118 (1.7%)	2/73 (2.7%)	1.000
Gastrointestinal	0/83 (0.0%)	2/118 (1.7%)	3/73 (4.1%)	0.135
Testicular	1/83 (1.2%)	3/118 (2.5%)	0/73 (0.0%)	0.476
**Imaging**				
**MRI performed**	**29/73 (39.7%)**	**112/118 (94.9%)**	**68/70 (97.1%)**	**<0.001**
Stalk thickening	5/13 (38.5%)	50/112 (44.6%)	29/70 (41.4%)	0.860
Sellar/suprasellar mass	8/13 (61.5%)	37/112 (33.0%)	26/70 (37.1%)	0.131
Mass >10mm	6/13 (46.2%)	13/73 (17.8%)	14/61 (23.0%)	0.078
**Endocrine**				
GH deficiency	22/39 (56.4%)	43/93 (46.2%)	23/59 (39.0%)	0.238
TSH deficiency	25/41 (61.0%)	62/99 (62.6%)	44/66 (66.7%)	0.806
ACTH deficiency	24/40 (60.0%)	47/91 (51.6%)	31/62 (50.0%)	0.584
FSH/LH deficiency	28/41 (68.3%)	76/96 (79.2%)	45/64 (70.3%)	0.289
Hyperprolactinemia	20/38 (52.6%)	56/95 (58.9%)	39/65 (60.0%)	0.744
Panhypopituitarism (all 5 axes)	6/83 (7.2%)	20/118 (16.9%)	13/73 (17.8%)	0.090
**CSF**				
Pleocytosis	16/29 (55.2%)	15/25 (60.0%)	13/20 (65.0%)	0.787
Elevated CSF protein	20/28 (71.4%)	15/25 (60.0%)	16/21 (76.2%)	0.466
Low CSF glucose	3/28 (10.7%)	4/21 (19.0%)	5/19 (26.3%)	0.409
CSF ACE elevated	2/2 (100.0%)	5/11 (45.5%)	4/12 (33.3%)	0.244
**Oligoclonal bands**	**0/29 (0.0%)**	**5/25 (20.0%)**	**3/20 (15.0%)**	**0.035**
**Serum markers**				
Serum ACE elevated	7/11 (63.6%)	20/42 (47.6%)	8/22 (36.4%)	0.329
IL-2R elevated	NA	8/11 (72.7%)	2/3 (66.7%)	1.000
Hypercalcemia	1/83 (1.2%)	6/118 (5.1%)	2/73 (2.7%)	0.290

**Continuous labs**				
CSF WBC count	24.0 (8.2-67.2), n=18	30.0 (14.2-52.2), n=12	11.5 (6.2-18.2), n=6	0.482
CSF protein	100.0 (57.5-197.0), n=23	120.0 (82.5-220.5), n=11	155.7 (90.5-249.0), n=7	0.630
Serum ACE value	73.4 (61.0-110.0), n=6	42.5 (22.0-120.8), n=26	72.6 (39.2-92.0), n=10	0.395
IL-2R value	NA	1108.0 (550.5-1767.0), n=11	1152.0 (828.5-2935.5), n=3	NA
ESR	33.0 (29.2-47.5), n=10	39.0 (30.2-59.5), n=12	49.0 (39.0-55.0), n=7	0.473
CRP	NA	2.0 (1.4-23.6), n=7	22.0 (17.0-29.0), n=5	NA
**Follow-up (months)**	**49.0 (19.0-70.0), n=50**	**36.0 (17.2-96.0), n=78**	**9.0 (3.0-36.0), n=28**	**0.003**
**Recurrence**				
**Overall recurrence**	**31/59 (52.5%)**	**46/97 (47.4%)**	**16/60 (26.7%)**	**0.009**
Recurrence type - neurological	28/31 (90.3%)	44/46 (95.7%)	16/16 (100.0%)	0.436
**Maintenance Therapy**				
**Hormonal replacement therapy**	**20/41 (48.8%)**	**25/93 (26.9%)**	**18/40 (45.0%)**	**0.022**
Desmopressin	15/41 (36.6%)	22/93 (23.7%)	16/40 (40.0%)	0.107
**Corticosteroids**	**36/41 (87.8%)**	**83/88 (94.3%)**	**30/39 (76.9%)**	**0.016**
Methotrexate	0/39 (0.0%)	28/93 (30.1%)	11/40 (27.5%)	<0.001
**Infliximab**	**0/41 (0.0%)**	**11/93 (11.8%)**	**7/40 (17.5%)**	**0.028**
Mycophenolate mofetil	0/41 (0.0%)	12/93 (12.9%)	5/40 (12.5%)	0.062
Azathioprine	0/41 (0.0%)	11/93 (11.8%)	4/40 (10.0%)	0.073
Cyclophosphamide	0/41 (0.0%)	6/93 (6.5%)	0/40 (0.0%)	0.080
Cyclosporine	0/41 (0.0%)	5/93 (5.4%)	0/40 (0.0%)	0.078
Radiotherapy	2/41 (4.9%)	2/93 (2.2%)	0/40 (0.0%)	0.362
Rituximab	0/41 (0.0%)	0/93 (0.0%)	1/40 (2.5%)	0.232

5. References

1. Ren STJ, Boakye H. A RARE CASE OF TRANSIENT ARGININE VASOPRESSIN DEFICIENCY CAUSED BY NEUROSARCOIDOSIS. Chest. 2024 Oct;166(4 Suppl):A3184.
2. Anwar A, Aleem M. A RARE CASE OF NEUROSARCOIDOSIS MIMICKING MALIGNANT MELANOMA-ASSOCIATED CARCINOMATOUS MENINGITIS. Chest. 2024 Oct;166(4 Suppl):A3215. doi:10.1016/j.chest.2024.06.1694.
3. Razia D, Khokar AI, Saeed AI. IT'S NOT MENINGITIS: SAMPLING OF MEDIASTINAL LYMPHADENOPATHY LED TO DIAGNOSIS OF NEUROSARCOIDOSIS. Chest. 2024 Oct;166(4 Suppl):A4481. doi:10.1016/j.chest.2024.06.3.
4. Kothari V, Yavuz S. New Onset Hypopituitarism with Disseminated Brain Lesions Causing Central DI & Hypogonadism. Endocr Pract. 2024 May;30(5):S206. doi:10.1016/j.eprac.2024.03.353.
5. Vander Heyde A, Gysbrechts C, Van der Biest AM. Systemic sarcoidosis presenting with hypercalcemia, sinusitis and hypothalamic-pituitary dysfunction. Acta Clin Belg. 2024;79(3):229–233. doi:10.1080/17843286.2024.2366714.
6. Badini KU, Ram N. Case Report of an Elderly Woman With Neurosarcoidosis. Endocr Pract. 2024;30(5):S206. doi:10.1016/j.eprac.2023.10.071.
7. Desmukh P, Shaikh S, Desmukh V. An unusual case of isolated pituitary sarcoidosis presenting as panhypopituitarism due to empty sella syndrome. Indian J Endocrinol Metab. 2023;27(Suppl 1):S350.
8. Niedzialkowska EM, Blazin T, Strubchevska K, Kozyk M, Saini N. Neurosarcoidosis Presenting With Panhypopituitarism. J Endocr Soc. 2023 Nov 1;7(Suppl 1):A724. doi:10.1210/jendso/bvad114.1357.
9. Galiana Rodriguez Caballero MG, Romero F, Escurra C, Rodriguez R, Rolon R, Valinotti E, Ayala A. Granulomatous Hypophysitis With Magnificent Response To Glucocorticoids. J Endocr Soc. 2023 Nov 1;7(Suppl 1):A675. doi:10.1210/jendso/bvad114.1263.
10. Alfares K, Han HJ, Athimulam S. Neurosarcoidosis Induced Panhypopituitarism. J Endocr Soc. 2023 Nov 1;7(Suppl 1):A677–A678. doi:10.1210/jendso/bvad114.1268.
11. Theodoropoulou E, Chelioti E, Palamaris K, Christofilidis G, Kaniadaki M, Stofas A, Papadaki M, Gakiopoulou H. Sarcoidosis and COVID-19: is there a link? [abstract E-PS-16-013]. Virchows Arch. 2023 Sep;483(Suppl 1):S322. doi:10.1007/s00428-023-03602-w.
12. Forrest A. An Unusual Case of Central Adrenal Insufficiency Following the Diagnosis of Presumed Neuro-Sarcoidosis. Journal of the Endocrine Society. 2022 Nov;6(Suppl 1):A111. doi:10.1210/jendso/bvac150.226.
13. Siddiqui M, Yasmeen T. Panhypopituitarism Secondary to Neurosarcoidosis with Superimposed Mycobacterium Nebraskense Meningitis [abstract #1185035]. Endocr Pract. 2022;28(5 Suppl):S105–S106. doi:10.1016/j.eprac.2022.03.253.
14. Kennedy SE, Jung F, Hadigal S, Patel DC, Gomez Manjarres DC. Frequent Falls - An Unusual Presentation of Sarcoidosis. Am J Respir Crit Care Med. 2022;205:A4068.
15. Bjorling A, Kolia NR. Primary Presentation of Neurosarcoidosis as a Pituitary Mass with Sinonasal Involvement. J Neurol Surg B Skull Base. 2022;83(S01):S1–S270. doi:10.1055/s-0042-1743906.
16. Bengtsen MB, Rasmussen TK, Nørgaard LJ. Neurosarkoidose i hypofysen. Ugeskr Læger. 2021 May 17;183(20):V02210159.
17. Venugopal S, Neupane B, Nepal M, Chavez LF. Unmasking of Neurosarcoidosis. J Endocr Soc. 2021 May 3;5(Suppl 1):A618. doi:10.1210/jendso/bvab048.1262.
18. Afreen S, Mittal A, Hasan F. Neurosarcoidosis and the Pituitary. J Endocr Soc. 2021 May 3;5(Suppl 1):A593. doi:10.1210/jendso/bvab048.1211.

19. Holmes J. DYSPNEA AND HYPOPITUITARISM: A UNIFYING DIAGNOSIS. JGIM. 2020;35(Suppl 1):S393.
20. Rana S. NOW YOU SEE IT, NOW YOU DON'T: A CASE OF DISAPPEARING DIABETES INSIPIDUS. Journal of the Endocrine Society. 2019 Apr-May;3(Suppl 1):Abstract #805794. doi:10.1210/js.2019-abstract-805794.
21. Matos T, Costa C, Vale Sd, Bugalho MJ. SAT-237 Isolated Hypogonadotropic Hypogonadism in a Male with Sarcoidosis [abstract SAT-237]. Journal of the Endocrine Society. 2020 May;4(Suppl 1):A489–A490. doi:10.1210/jendso/bvaa046.964.
22. Shah MS, Oo YH. SUN-297 Brain Fog: An Important Cue to Neurosarcoidosis and Its Flare-Up? [abstract SUN-297]. Journal of the Endocrine Society. 2020 May;4(Suppl 1):A645. doi:10.1210/jendso/bvaa046.1260.
23. Saini KK, Ankireddypalli A, Marx T, Venkatraman GK. M109. Intramedullary Spinal Sarcoidosis with Cauda Equina Syndrome and Panhypopituitarism: A Rare Case of Isolated Neurosarcoidosis Complicated by Varicella Zoster Encephalitis [abstract M109]. Annals of Neurology. 2019 Oct;86(Suppl 24):S232–S233.
24. Paramasivan AM, Buddhdev K, Drincic A. SUN-406 Complete Recovery of Central Diabetes Insipidus Associated with Isolated Stalk Thickening in a Neurosarcoidosis Patient. Journal of the Endocrine Society. 2019 Apr-May;3(Suppl 1):SUN-406. doi:10.1210/js.2019-SUN-406.
25. O'Connor A, Gilmore D, Shaamile F, McQuaid S. SUN-427 Hypopituitarism as a Rare Manifestation in a Patient with Neurosarcoidosis. Journal of the Endocrine Society. 2019 Apr-May;3(Suppl 1):SUN-427. doi:10.1210/js.2019-SUN-427.
26. Saini KK, Capitle E, Laharwal M. SAT-LB082 Neurosarcoidosis Presenting as Panhypopituitarism, Diabetes Insipidus and Sellar Mass. Journal of the Endocrine Society. 2019 Apr-May;3(Suppl 1):SAT-LB082. doi:10.1210/js.2019-SAT-LB082.
27. Ferreira A, Bastos F, Nunes da Silva T, Cordeiro M, Portugal J. SUN-428 Neurosarcoidosis: A Rare Cause of Hypothalamic-Pituitary Dysfunction. Journal of the Endocrine Society. 2019 Apr-May;3(Suppl 1):SUN-428. doi:10.1210/js.2019-SUN-428.
28. Wang F, Yazdani S, Dong J, Xiong W. Neurogenic Shock: A Rare Presentation of Neurosarcoidosis. Neurocritical Care. 2019;31(Suppl 1):S306. Poster 296.
29. Jalil B, Gearhart A, El-Kersh K. 732: Sarcoid Meningitis: An Extremely Rare Cause of Acute Encephalopathy [abstract 732]. Critical Care Medicine. 2018 Jan;46(1 Suppl):353. doi:10.1097/01.ccm.0000528761.64500.22.
30. Desai N, Khadilkar S, Patel B. Hypertrophic Cranial Polyneuropathies and Pituitary Lesions (P4.051). Neurology. 2017 Apr 18;88(16 Suppl):P4.051.
31. Mitrofanova L, Ryazanov P, Antonova I. A rare case of pituitary sarcoidosis. Virchows Arch. 2016;469(Suppl 1):S346.
32. Campos Costa E, Pereira AP, Marques IR, Ribeiro AC. P12173 Neurosarcoidosis: sometimes, the essential is invisible to the eye [abstract P12173]. Eur J Neurol. 2020 Jun;27(Suppl 1):944. doi:10.1111/ene.14307.
33. Doehring J, Moscona J. WATER YOU LOOKING FOR? PAN-HYPO-PITUITARISM IN NEUROSARCOIDOSIS [abstract]. J Gen Intern Med. 2016 May;31(Suppl 2):S782. doi:10.1007/s11606-016-3657-3.
34. Ho G, Brizzi K, Meredith D, Laws E, Berkowitz A. A Case of Primary Neurosarcoid Presenting as Foster-Kennedy Syndrome with Diabetes Insipidus (P6.146). Neurology. 2016 Apr 5;86(16 Suppl):P6.146.
35. Boer M, Lapauw B. Tiredness can be life-threatening. Acta Clin Belg. 2016;71(Suppl 3):3.
36. Sanghi V, Kapoor A. Neurosarcoidosis induced panhypopituitarism. Crit Care Med. 2015 Dec;43(12 Suppl):1260. doi:10.1097/01.ccm.0000474121.26732.1d.
37. Krenzlin H, Jussen D, Musahl C, Scheil-Bertram S, Wernecke K, Horn P. A Rare Case of Isolated Cerebral Sarcoidosis Presenting as Suprasellar Mass Lesion with Salt-Wasting Hypopituitarism. Neurol Surg Rep. 2015 Dec;76(2):e140–5. doi:10.1055/s-0035-1555120 PubMed PMID: 26649231.
38. Resende E, Caldeira M, Freitas E, Sá M, Ferreira M, Abreu S. Diabetes insípida... um indício para o diagnóstico de sarcoidose. Rev Port Endocrinol Diabetes Metab. 2014 Jul-Dec;9(2):144–8. doi:10.1016/j.rpedm.2014.06.002.

39. Leoni-Moreno JC, Venkatachalam K, Safford R. An unlikely unifying diagnosis of neuro and cardiac sarcoidosis. J Am Coll Cardiol. 2014 Apr 1;63(12 Suppl):A672. doi:10.1016/S0735-1097(14)60672-0.
40. Williamson A, Weaver A, Kandavar R. PANHYPOPITUITARISM, SARCOIDOSIS, AND AML: SIMULTANEOUS STORM OR COMPLEX CASCADE? [abstract]. J Gen Intern Med. 2014 Apr;29(Suppl 1):S405. doi:10.1007/s11606-014-2831-y.
41. Murphy KP, Kennedy MP, Barry JE, O'Regan KN, Power DG. New-Onset Mediastinal and Central Nervous System Sarcoidosis in a Patient with Metastatic Melanoma Undergoing CTLA4 Monoclonal Antibody Treatment. Oncol Res Treat. 2014;37(6):351–3. doi:10.1159/000362614 PubMed PMID: 24943333.
42. Hwang JK, Cho JH, Park SY, Son JI, Jo U, Chin SO, Lee YJ, Choi MC, Rhee SY, Kim EJ, Chon S. A Case of Possible Neurosarcoidosis Presenting as Intractable Headache and Panhypopituitarism. Case Rep Endocrinol. 2013;2013:816236. doi:10.1155/2013/816236 PubMed PMID: 23984120.
43. Sponholz Araujo M, Antunes Silva Pereira D, Guedes Baldi B, Salim Gonçalves Freitas C, Madeira Padovesi C, Adib Kairalla R, Roberto Ribeiro Carvalho C. Unusual multisystemic relapse of sarcoidosis after corticosteroids withdrawal. Eur Respir J. 2013;42(Suppl 57):P3805.
44. Cicognani C, Spinucci G, Riguzzi P, Nassetti S, Michelucci R, Zaccaroni S. Central nervous system involvement in sarcoidosis: are corticosteroids sufficient as initial treatment? [abstract]. Neurological Sciences. 2017 Oct;38(Suppl 1):S180. doi:10.1007/s10072-017-3023-3.
45. Alassil S, Naber T, Kouli W. Unmasking Pre-existant Central Diabetes Insipidus (CDI) in a Patient With Neurosarcoidosis After Gastric Bypass Surgery: A Rare Presentation [abstract 12]. Am J Kidney Dis. 2013 Apr;61(4):A12. doi:10.1053/j.ajkd.2013.02.046.
46. Linek JA, Morgenthau AS. Empty Sella Syndrome In A Patient With Sarcoidosis. Am J Respir Crit Care Med. 2012;185:A2961.
47. Langrand C, Bihan H, Raverot G, Varron L, Androdias G, Borson-Chazot F, Brue T, Cathebras P, Pinede L, Muller G, Broussolle C, Cotton F, Valeyre D, Seve P. Hypothalamo-pituitary sarcoidosis: a multicenter study of 24 patients. QJM. 2012 Nov;105(11):981–95. doi:10.1093/qjmed/hcs121 PubMed PMID: 22753232.
48. Matas A, Veiga A, Costa A, Guimarães P, Gabriel JP, Silva MR. P2691 NEUROSARCOIDOSIS WITHOUT SYSTEMIC CLINICAL FINDINGS [abstract P2691]. Eur J Neurol. 2020 Jun;27(Suppl 1):1018. doi:10.1111/ene.14307.
49. Thonke S, Abruscato M, Bende C, Baas H. Neurosarcoidosis presenting with stroke and cranial neuropathy: case report and neuroradiologic findings. Cerebrovasc Dis. 2011;31(suppl 2):168.
50. Coeytaux A, Emond H, Horvath J, Kurian M, Chofflon M, Landis T, Perrig S. P2752 Narcolepsy in neurosarcoidosis: a report of two cases [abstract P2752]. Eur J Neurol. 2020 Jun;27(Suppl 1):1031. doi:10.1111/ene.14307.
51. Gilbert J, Oguntolu V, Manawadu D, Salisbury J. Granulomatous infiltration of the pituitary gland. Endocrine Abstracts. 2010;21:P57.
52. Elamin M, Chalissery AJ, Keogan M, Farrell M, Brett F, Moroney JT. P263 Refractory neurosarcoidosis responding to infliximab [abstract P263]. Eur J Neurol. 2020 Jun;27(Suppl 1):767. doi:10.1111/ene.14307.
53. Bullmann C, Faust M, Hoffmann A, Heppner C, Jockenhövel F, Müller-Wieland D, Krone W. Five cases with central diabetes insipidus and hypogonadism as first presentation of neurosarcoidosis. Eur J Endocrinol. 2000 Apr;142(4):365–72. doi:10.1530/eje.0.1420365 PubMed PMID: 10754476.
54. Ingestad R, Stigmar G. Sarcoidosis with ocular and hypothalamic-pituitary manifestations. Acta Ophthalmol (Copenh). 1971;49(1):1–10. doi:10.1111/j.1755-3768.1971.tb02941.x PubMed PMID: 5122828.
55. Owen TK, Henneman J. Diffuse sarcoidosis associated with hypopituitarism and terminal renal failure. Br Med J. 1954 Mar 27;1(4864):736–7. doi:10.1136/bmj.1.4864.736.
56. Ach T, Ben Yahia W, Halloul I, Sghaier F, Atig A. Neurosarcoidosis-Induced Hypophysitis Mimicking Pituitary Macroadenoma. Cureus. 2023 Jun 2;15(6):e39865. doi:10.7759/cureus.39865 PubMed PMID: 37402929.

57. Guziejko K, Minarowski Ł, Piłaszewicz-Puza A, Szumera-Ciećkiewicz A, Mróz RM. Multiorgan sarcoidosis as a diabetes insipidus mask. Endocrinol Diabetes Metab Case Rep. 2022 Nov 1;2022:22-0266. doi:10.1530/EDM-22-0266 PubMed PMID: 4226.
58. Stuart CA, Neelon FA, Lebovitz HE. Disordered control of thirst in hypothalamic-pituitary sarcoidosis. N Engl J Med. 1980 Nov 6;303(19):1078–82. doi:10.1056/NEJM198011063031901 PubMed PMID: 6497.
59. Niedzialkowska E, Blazin T, Shelden D, Buras ED. Neurosarcoidosis With Panhypopituitarism: Two Cases and Literature Review. JCEM Case Reports. 2024 Aug;2(8):luae141. doi:10.1210/jcemcr/luae141.
60. Heard S, Machado FM, Ponnusamy JJ. Neurosarcoidosis Presenting With Confusion and Speech Alteration. Cureus. 2023 Jul 28;15(7):e42627. doi:10.7759/cureus.42627 PubMed PMID: 5256.
61. Kinoshita Y, Taguchi A, Yamasaki F, Nakamori M, Amatya VJ, Iida K. Isolated Neurohypophysial Sarcoidosis Involving the Cavernous Sinus Mimicking a Malignant Tumor. NMC Case Rep J. 2022 Jan 28;9:31–35. doi:10.2176/jns-nmc.2021-0296.
62. Gizzi MS, Lidov M, Rosenbaum D. Neurosarcoidosis presenting as a tumour of the basal ganglia and brainstem: sequential MRI. Neurol Res. 1993;15(2):93–96. doi:10.1080/01616412.1993.11740116.
63. Weber A, Behrend H, Gerdes H. Hypophysenvorderlappeninsuffizienz bei Sarkoidose. Dtsch Med Wochenschr. 1968;93(30):1428–1433.
64. Bledsoe LD, Cunningham DS. Isolated Galactorrhea in Sarcoidosis: A Case Report. Mil Med. 1994 Aug;159(8):584–5.
65. Younas MT, Dale J, Khan M. Progressive Neurosarcoidosis Presenting as Idiopathic Hypopituitarism With Atypical Radiological Features for 13 Years. Cureus. 2024 Nov 13;16(11):e73575. doi:10.7759/cureus.73575 PubMed PMID: 39545120.
66. Johns CE, Johns CS. A Case of Central Diabetes Insipidus Secondary to Neurosarcoidosis. Cureus. 2020 Nov 6;12(11):e11119. doi:10.7759/cureus.11119 PubMed PMID: 33304720.
67. Solis JG, Lugo AO, Florido MAR, Bonilla BAS, Rangel JM. Neurosarcoidosis Presentation as Adipsic Diabetes Insipidus Secondary to a Pituitary Stalk Lesion and Association with Anti-NMDA Receptor Antibodies. Case Rep Neurol Med. 2020 Jun 25;2020:7956350. doi:10.1155/2020/7956350 PubMed PMID: 32655120.
68. Vanhoof J, Wilms G, Bouillon R. Hypothalamisch Hypopituitarisms Met Hyperfagie En Subacute Dementie Op Basis Van Neurosarcoidose: Een Geval en Literatuuroverzicht. Acta Clin Belg. 1992 Oct;47(5):319–28. doi:10.1080/17843286.1992.11718250 PubMed PMID: 1475120.
69. Berntsson SG, Elmgren A, Gudjonsson O, Grabowska A, Landtblom AM, Moraes-Fontes MF. A comprehensive diagnostic approach in suspected neurosarcoidosis. Sci Rep. 2023 Apr 19;13(1):6539. doi:10.1038/s41598-023-33631-z PubMed PMID: 37077120.
70. Clément J, Dubois de Mont-Marin A. Sarcoidosis of the pituitary gland and stalk in a man presenting asthenia, impotence, loss of libido, polyuria, and polydipsia. Lancet. 2024 Dec 14;404(10470):2460–1. doi:10.1016/S0140-6736(24)02148-3 PubMed PMID: 39671120.
71. Carbone RG, Penna D, Monselise A, Puppo F. Systemic sarcoidosis with pituitary adenoma. J Clin Imaging Sci. 2022 Jun 10;12:43. doi:10.25259/JCIS_43_2022 PubMed PMID: 35795410.
72. Thota G, Gribkova Y, Desai RN. Neurosarcoidosis From Pituitary Expansion to Empty Sella. J Urgent Care Med. 2023;17(8):29–32.
73. Balcha TE, Woldeyohannes AM, Aboye AG. Imaging Findings of Concomitant Pulmonary and Central Nervous System Sarcoidosis: A Case Report. Ethiop J Health Sci. 2022 May;32(3):661–4. doi:10.4314/ejhs.v32i3.23 PubMed PMID: 35695420.
74. Padiyar S, Doley D, Babu R, Mathew J. An Unusual Case of Vomiting and Arthritis: A Diagnostic Enigma. J Clin Rheumatol. 2021 Oct 22. doi:10.1097/RHU.0000000000001712.

75. Borawski B, Kuca P, Zieliński G, Maksymowicz M, Witek P. Pathologically-confirmed isolated hypothalamo-pituitary sarcoidosis refractory to pulse-dose glucocorticoids and successfully treated with methotrexate. Anaesthesiol Intensive Ther. 2022. doi:10.4183/acb.2022.244.
76. Ukhanova YA, Ilovayskaya IA, Terpigorev SA. Panhypopituitarism as the first manifestation of sarcoidosis: case report [abstract]. Endocrine Abstracts. 2017;49:EP1056. doi:10.1530/endoabs.49.EP1056.
77. Chen X, Chen G, Fu Z, Li Q. 18F-FDG PET/CT Findings in a Patient With Neurosarcoidosis. Clin Nucl Med. 2020;45(8):640-641.
78. Kakehi E, Adachi S, Fukuyasu Y, Hashimoto Y, Sakurai S, Hirotani A, Danbara H, Shimizu K, Fujita R, Teraura H, Kotani K, Matsumura M. Magnetic resonance imaging and symptoms in patients with neurosarcoidosis and central diabetes insipidus. Endokrynol Pol. 2019;70(5):430–7. doi:10.5603/EP.a2019.0035 PubMed PMID: 31642491.
79. Cognat E, Roriz M, Chabriat H, Buffon-Porcher F. Neurosarcoidosis: leptomeninigeal, cavernous sinus and pituitary involvement. QJM. 2017 Jun 1;110(6):397–8. doi:10.1093/qjmed/hcx037 PubMed PMID: 28339675.
80. Hassani FD, Fadli M, El Abbadi N. Sarcoïdose hypophysaire mimant un adénome hypophysaire: à propos d'un cas et revue de la littérature. Pan Afr Med J. 2019 Jun 7;33:104. doi:10.11604/pamj.2019.33.104.17849.
81. Abushamat LA, Kerr JM, Lopes MBS, Kleinschmidt-DeMasters BK. Very Unusual Sellar/Suprasellar Region Masses: A Review. J Neuropathol Exp Neurol. 2019 Aug 1;78(8):673–84. doi:10.1093/jnen/nlz044 PubMed PMID: 31321422.
82. Oye M, Chahin M, Krishnan N, Reddy P. Neurosarcoidosis manifesting as panhypopituitarism. Clin Case Rep. 2019 Sep 27;7(11):2251–2. doi:10.1002/ccr3.2521 PubMed PMID: 31754407.
83. de Waard WIQ, van Battum PLH, Mostard RLM. Central diabetes insipidus due to sarcoidosis. Sarcoidosis Vasc Diffuse Lung Dis. 2017;34(2):191–193.
84. Merinopoulos D, Hayes F, Gallagher DA, Dasgupta B. A case report of neurosarcoidosis successfully treated with an infliximab biosimilar after a relapse while on dual therapy. Rheumatology (Oxford). 2017;56(11):2044–2046.
85. Alsahwi N, Blavo D, Karanchi H. Systemic sarcoidosis with hypercalcaemia, hypothalamic-pituitary dysfunction and thyroid involvement. BMJ Case Rep. 2016 Oct 17;2016:bcr2016216503.
86. Aghajanova L, Jaffe RB, Herndon CN. Infiltrative Neurosarcoidosis Presenting as Secondary Amenorrhea: Case Report and Review of the Literature. Obstet Gynecol Surv. 2013 Jun;68(6):449–455.
87. Prayson RA. Biopsy proven pituitary sarcoidosis presenting as a possible adenoma. J Clin Neurosci. 2016;31:188–190.
88. Tanaka K, Yamamoto M, Okazaki K, Yamaguchi T, Sugimoto T. Partial Improvement of Anterior Pituitary Deficiency Following Steroid Treatment in a Patient with Neurosarcoidosis Accompanied by Central Diabetes Insipidus. Intern Med. 2013;52(15):1699–1703.
89. Sanghi V, Kapoor A. Unusual Presentation of Central Diabetes Insipidus in a Patient With Neurosarcoidosis. J Investig Med High Impact Case Rep. 2016;4(3):2324709616667511.
90. Bihan H, Guillot H, Fysekidis M, Cohen R, Gille T, Nunes H, et al. Sarcoïdose : l'atteinte des hormones à dépendance antéhypophysaire est insuffisamment diagnostiquée. Rev Med Interne. 2012;33(8):439-444.
91. O'Reilly MW, Sexton DJ, Dennedy MC, Counihan TJ, Finucane FM, O'Brien T, et al. Radiological remission and recovery of thirst appreciation after infliximab therapy in adipsic diabetes insipidus secondary to neurosarcoidosis. QJM. 2013;106(6):569–572.
92. Marko NF, Prayson RA, Weil RJ. Sellar mass with vision loss and hypopituitarism. J Clin Neurosci. 2012;19(9):1282.

93. Anthony J, Esper GJ, Ioachimescu A. Hypothalamic-pituitary sarcoidosis with vision loss and hypopituitarism: case series and literature review. Pituitary. 2016 Feb;19(1):19–29. doi:10.1007/s11102-015-0678-x PubMed PMID: 26343277.
94. Bihan H, Christozova V, Dumas JL, Jomaa R, Valeyre D, Tazi A, Reach G, Krivitzky A, Cohen R. Sarcoidosis: Clinical, Hormonal, and Magnetic Resonance Imaging (MRI) Manifestations of Hypothalamic-Pituitary Disease in 9 Patients and Review of the Literature. Medicine (Baltimore). 2007 Sep;86(5):259–68. doi:10.1097/MD.0b013e318156ae8c PubMed PMID: 17873755.
95. Miyoshi T, Otsuka F, Takeda M, Inagaki K, Otani H, Ogura T, Ichiki K, Amano T, Makino H. An Elderly Patient with Sarcoidosis Manifesting Panhypopituitarism with Central Diabetes Insipidus. Endocr J. 2007 Jun;54(3):425–30. doi:10.1507/endocrj.K06-121 PubMed PMID: 17485856.
96. Pillai P, Ray-Chaudhury B, Ammirati M, Chiocca EA. Solitary pituitary sarcoidosis with normal endocrine function: case report. J Neurosurg. 2008 Mar;108(3):591–4. doi:10.3171/JNS/2008/108/3/0591 PubMed PMID: 18312110.
97. Akakın A, Konya D, Akakın D, Kılıç T. Isolated Pituitary Neurosarcoidosis: A Case Report and Review of the Literature. Marmara Med J. 2011 Oct;24(3):196–9. doi:10.5472/MMJ.2011.02028.1.
98. Maldonado-Morales ML, Ruiz-Calvo A, Santos-Bueso E, Del-Hierro A, López-Abad C, Díaz-Valle D, Benítez-del-Castillo JM, García-Sánchez J. Visual fields defect as initial presentation of neurosarcoidosis. Arch Soc Esp Oftalmol. 2012 Feb;87(2):50–4. doi:10.1016/j.oftal.2011.06.012 PubMed PMID: 22284419.
99. Ogawa Y, Tominaga T, Ikeda H. Neurosarcoidosis Manifesting as Tremor of the Extremities and Severe Hypopituitarism: Case Report. Neurol Med Chir (Tokyo). 2008 Jul;48(7):314–7. doi:10.2176/nmc.48.314 PubMed PMID: 18653982.
100. Luthra M, Siegel RE. A Cool Case of Hypothermia. Intern Med. 2010;49(14):1455. doi:10.2169/internalmedicine.49.3720 PubMed PMID: 20647614.
101. Bakar EE, Selçuki D. Nörosarkoidoz: hipofiziyer-hipotalamik tutulumlu bir olgu sunumu. Gülhane Tıp Derg. 2009;51:247–250.
102. Tahir MZ, Shaikh F, Siddiqui AA. Primary chiasmal sarcoid granuloma masquerading as glioma of the optic chiasm. J Coll Physicians Surg Pak. 2010 Oct;20(10):695–6. PubMed PMID: 20959050.
103. Motta M, Alongi F, Bolognesi A, Cozzarini C, Di Muzio N, Fazio F. Remission of Refractory Neurosarcoidosis Treated With Brain Radiotherapy: A Case Report and a Literature Review. J Investig Med High Impact Case Rep. 2013;1(1):2324709613481230.
104. Sundaresan P, Jayamohan J. Stereotactic radiotherapy for the treatment of neurosarcoidosis involving the pituitary gland and hypothalamus. J Med Imaging Radiat Oncol. 2009 Dec;52(6):622–626.
105. Titlić M, Dolić K, Bešenski N. Mild cognitive disorder as clinical manifestation of pituitary stalk neurosarcoidosis: case report. Acta Clin Croat. 2011;50(4):581–587.
106. Asabella AN, Gatti P, Notaristefano A, Iuele F, Altini C, Rubini G. F-18 FDG PET/CT in the Diagnosis of a Rare Case of Neurosarcoidosis in a Patient With Diabetes Insipidus. Clin Nucl Med. 2011;36(9):795–797.
107. Afshar K, Engelfried K, Sharma OP. Sarcoidosis: a rare cause of Kleine-Levin-Critchley syndrome. Sarcoidosis Vasc Diffuse Lung Dis. 2008;25(1):60–63.
108. Kimball MM, Wind JJ, Codispoti K-ET, Jones RV, Leiphart JW. Neurosarcoidosis presenting as an isolated intrasellar mass: case report and review of the literature. Clin Neuropathol. 2010;29(3):156–162.
109. Yoshioka K, Tanaka N, Yamagami K, Inoue T, Hosoi M. Arginine Vasopressin-Independent Mechanism of Impaired Water Excretion in a Patient with Sarcoidosis Complicated by Central Diabetes Insipidus and Glucocorticoid Deficiency. Case Rep Med. 2011;2011:145856.
110. Alam T, Thomas S. Diabetes insipidus secondary to sarcoidosis presenting with caseating granuloma. BMJ Case Rep. 2011;2011:bcr0220113840.

111. Inaba H, Suzuki S, Shigematsu S, Kobayash S, Nishio S, Hashizume K. Spontaneous Remission of Diabetes Insipidus Due to CNS Sarcoidosis. Intern Med. 2009;48(4):225–229.
112. Skowasch D, Pabst S, Wilhelm K, Grohé C. Diabetes insipidus bei Neurosarkoidose. Pneumologie. 2011;65(08):496–497. doi.org/10.1055/s-0030-1256374.
113. Hosseini AA, Viswanath A, El-Lahawi MK. Neurosarcoidosis presenting with persistent vomiting. Neurosciences (Riyadh). 2007 Jul;12(3):250–3. PubMed PMID: 24045579.
114. Molina A, Mañá J, Villabona C, Fernández-Castañer M, Soler J. Hypothalamic-Pituitary Sarcoidosis with Hypopituitarism: Long-Term Remission with Methylprednisolone Pulse Therapy. Pituitary. 2002 May;5(1):33–6. doi:10.1023/A:1025482312683 PubMed PMID: 12903964.
115. Iwai H, Keike R, Hagiyama H, Nagasaka K, Koga M, Nonomura Y, et al. A case of sarcoidosis characterized by a nasopharyngeal tumor and neurological lesions. Jpn J Clin Immunol. 2001;24(1):21–28.
116. Lanzi R, Previtali SC, Sansone V, Scavini M, Fortunato L, Gatti E, Meola G, Bosi E, Losa M. Hypokalemic periodic paralysis in a patient with acquired growth hormone deficiency. J Endocrinol Invest. 2007 Apr;30(4):341–5. doi:10.1007/BF03346299 PubMed PMID: 17551283.
117. Smith D, Cullen MJ. Two cases of hypothalamic-pituitary sarcoidosis. J R Soc Med. 2005;98:167–169.
118. Aide N, Benayoun M, Kerrou K, Khalil A, Cadranel J, Talbot JN. Impact of [18F]-fluorodeoxyglucose ([18F]-FDG) imaging in sarcoidosis: unsuspected neurosarcoidosis discovered by [18F]-FDG PET and early metabolic response to corticosteroid therapy. Br J Radiol. 2007 Jul;80(955):e67–71. doi:10.1259/bjr/67448858 PubMed PMID: 17704309.
119. Hattori Y, Hashimoto Y, Matsuoka R, Ohtsuka Y, Nakayama I, Honda Y, et al. [A case of sarcoidosis with diabetes insipidus and pituitary tumor]. Nihon Naika Gakkai Zasshi. 2001 Feb 10;90(2):326-328.
120. Gungor SH, Kiter G, Karasu D. Refractory neurosarcoidosis: a dramatic response to infliximab [letter]. Am J Med. 2004 Aug 1;117(3):277–8. doi:10.1016/j.amjmed.2004.03.023.
121. Randeva HS, Davison R, Chamoun V, Bouloux PMG. Isolated Neurosarcoidosis A Diagnostic Enigma: Case Report and Discussion. Endocrine. 2002 Apr;17(3):241–7. doi:10.1386/endo:17.3.241 PubMed PMID: 12088210.
122. Tabuena RP, Nagai S, Handa T, Shigematsu M, Hamada K, Ito I, Izumi T, Mishima M, Sharma OP. Diabetes Insipidus from Neurosarcoidosis: Long-term Follow-up for More than Eight Years. Intern Med. 2004 Oct;43(10):960–6. doi:10.2169/internalmedicine.43.960 PubMed PMID: 15575239.
123. Pujol Olivares X, Vilamú Talavera M, Esquerrà Alibés MT, Deniel Rosanas J. Sarcoidosis e hipopituitarismo. Aten Primaria. 2003;32(2):122.
124. Pitale SU, Camacho PM, Gordon DL. Central Nervous System Involvement in Sarcoidosis Presenting as a Recurrent Pituitary Mass. Endocrinologist. 2000 Sep;10(5):357–60. doi:10.1097/00019616-200010050-00011.
125. Murialdo G, Tamagno G. Endocrine aspects of neurosarcoidosis. J Endocrinol Invest. 2002 Jul-Aug;25(7):650–62. doi:10.1007/BF03345501 PubMed PMID: 12240909.
126. Rubin MR, Bruce JN, Khandji AG, Freda PU. Sarcoidosis within a Pituitary Adenoma. Pituitary. 2001 Dec;4(4):195–202. doi:10.1023/A:1021200132338 PubMed PMID: 12510950.
127. Vogl TJ, Stemmler J, Scriba PC, Vogl C, Lissner J. Sarcoidosis of the hypothalamus and pituitary stalk. Eur Radiol. 1992 Feb;2(1):76–8. doi:10.1007/BF00171228.
128. Loh KC, Green A, Dillon WP Jr, Fitzgerald PA, Weidner N, Tyrrell JB. Diabetes insipidus from sarcoidosis confined to the posterior pituitary. Eur J Endocrinol. 1997 Nov;137(5):514–9. doi:10.1530/eje.0.1370514 PubMed PMID: 9400287.
129. Case 10-1991: Presentation of Case. N Engl J Med. 1991 Mar 7;324(10):686.

130. Christoforidis GA, Spickler EM, Recio MV, Mehta BM. MR of CNS Sarcoidosis: Correlation of Imaging Features to Clinical Symptoms and Response to Treatment. AJNR Am J Neuroradiol. 1999 Apr;20(4):655–669.
131. Kim J, de Lotbiniere ACJ, Guoth MS, Brines ML. Neurosarcoidosis Presenting as Hypopituitarism and a Cystic Pituitary Mass. Am J Med Sci. 1998 Mar;315(3):220–4. doi:10.1097/00000441-199803000-00010 PubMed PMID: 9519114.
132. Bakshi R, Fenstermaker RA, Bates VE, Ravichandran TP, Goodloe S Jr, Kinkel WR. Neurosarcoidosis presenting as a large suprasellar mass: magnetic resonance imaging findings. Clin Imaging. 1998 Sep-Oct;22(5):322–6. doi:10.1016/S0899-7071(98)00032-4 PubMed PMID: 9794565.
133. Hidaka N, Takizawa H, Miyachi S, Hisatomi T, Kosuda T, Sato T. Case Report: A Case of Hypothalamic Sarcoidosis with Hypopituitarism and Prolonged Remission of Hypogonadism. Am J Med Sci. 1987 Nov;294(5):357–63. doi:10.1097/00000441-198711000-00010 PubMed PMID: 3425579.
134. Scott IA, Stocks AE, Saines N. Hypothalamic/pituitary sarcoidosis. Aust NZ J Med. 1987 Jun;17(3):243–5. doi:10.1111/j.1445-5994.1987.tb01217.x PubMed PMID: 3478013.
135. Mißler U, Mack M, Nowak G, Müller-Esch G, Reusche E, Borgis KJ, Löhrs U, Arnold H. Pituitary Sarcoidosis. Klin Wochenschr. 1990 Apr;68(7):342–5. doi:10.1007/BF01648873 PubMed PMID: 2341219.
136. Sato N, Sze G, Kim JH. Cystic Pituitary Mass in Neurosarcoidosis. AJNR Am J Neuroradiol. 1997 Jun-Jul;18(6):1182–5. PubMed PMID: 9202863.
137. Lewis R, Wilson J, Smith FW. Diabetes Insipidus Secondary to Intracranial Sarcoidosis Confirmed by Low-Field Magnetic Resonance Imaging. Magn Reson Med. 1987 Oct;5(4):466–70. doi:10.1002/mrm.1910050509 PubMed PMID: 3683163.
138. Lara Capellan JI, Cuellar Olmedo L, Martinez Martin J, Mar Marin MD, Garcia Villanueva M, Marin Zarza F, de la Calle Blasco H. Intrasellar mass with hypopituitarism as a manifestation of sarcoidosis: Case report. J Neurosurg. 1990 Aug;73(2):283–6. doi:10.3171/jns.1990.73.2.0283 PubMed PMID: 2366086.
139. Féry F, Plat L, van de Borne P, Cogan E, Mockel J. Impaired Counterregulation of Glucose in a Patient with Hypothalamic Sarcoidosis. N Engl J Med. 1999 Mar 18;340(11):852–6. doi:10.1056/NEJM199903183401105 PubMed PMID: 10080140.
140. Cannavò S, Romano C, Buffa R, Faglia G. Granulomatous sarcoidotic lesion of hypothalamic-pituitary region associated with Rathke's cleft cyst. J Endocrinol Invest. 1997 Feb;20(2):77–81. doi:10.1007/BF03347978 PubMed PMID: 9140788.
141. Arle JE, Judkins AR, Kotapka MJ. Neurosarcoidosis Presenting in the Pituitary Gland with Normal Endocrine Studies. Skull Base Surg. 1998;8(4):219–24. doi:10.1055/s-2008-1058579 PubMed PMID: 17171144.
142. Freda PU, Silverberg SJ, Post KD, Wardlaw SL. Hypothalamic-Pituitary Sarcoidosis. Trends Endocrinol Metab. 1992 Nov;3(9):321–5. doi:10.1016/1043-2760(92)90111-H PubMed PMID: 18407130.
143. Tohda S, Akagawa S, Miura Y, Satoh T, Murota N, Tanoue M, et al. [A case of sarcoidosis associated with diabetes insipidus, anterior pituitary insufficiency and alveolar hypoventilation on a central basis]. Nihon Naika Gakkai Zasshi. 1989;78(3):362-363.
144. Vesely DL. Hypothalamic Sarcoidosis: A New Cause of Morbid Obesity. South Med J. 1989 Jun;82(6):758–61. doi:10.1097/00007611-198906000-00019 PubMed PMID: 2734645.
145. Lawton FG, Beardwell CG, Shalet SM, Daws RA. Hypothalamic-pituitary disease as the sole manifestation of sarcoidosis. Postgrad Med J. 1982 Dec;58(686):771–2. doi:10.1136/pgmj.58.686.771 PubMed PMID: 7167435.
146. Lawrence WP, El Gammal T, Pool WH Jr, Apter L. Radiological manifestations of neurosarcoidosis: report of three cases and review of literature. Clin Radiol. 1974 Jul;25(3):343–8. doi:10.1016/s0009-9260(74)80164-5.

147. Stuart CA, Neelon FA, Lebovitz HE. Hypothalamic insufficiency: the cause of hypopituitarism in sarcoidosis. Ann Intern Med. 1978 May;88(5):589–94. doi:10.7326/0003-4819-88-5-589 PubMed PMID: 646252.
148. Selenkow HA, Tyler HR, Matson DD, Nelson DH. Hypopituitarism due to hypothalamic sarcoidosis. Am J Med Sci. 1959 Oct;238(4):456–63.
149. Chiang R, Marshall MC Jr, Rosman PM, Hotson G, Mannheimer E, Wallace EZ. Empty sella turcica in intracranial sarcoidosis: pituitary insufficiency, primary polydipsia, and changing neuroradiologic findings. Arch Neurol. 1984 Jun;41(6):662–5. doi:10.1001/archneur.1984.04050180092025 PubMed PMID: 6721715.
150. Schonell ME, Gillespie WJ, Maloney AF. Cerebral sarcoidosis. Brit J Dis Chest. 1968 Jul;62(3):195–9. doi:10.1016/s0007-0971(68)80016-x.
151. Shealy CN, Kahana L, Engel FL, McPherson HT. Hypothalamic-pituitary sarcoidosis: a report on four patients, one with prolonged remission of diabetes insipidus following steroid therapy. Am J Med. 1961 Jan;30(1):46–55. doi:10.1016/0002-9343(61)90064-0.
152. Nora JR, Levitsky JM, Zimmerman HJ. Sarcoidosis with panhypopituitarism and diabetes insipidus. Ann Intern Med. 1959 Dec;51(6):1400–9. doi:10.7326/0003-4819-51-6-1400.
153. Jawadi MH, Hanson TJ, Schemmel JE, Beck P, Katz FH. Hypothalamic Sarcoidosis and Hypopituitarism. Hormone Res. 1980;12(1):1–9. doi:10.1159/000179088.
154. Ludmerer KM, Kissane JM, eds. Visual impairment, pituitary dysfunction, and hilar adenopathy in a young man. Am J Med. 1986 Feb;80(2):259–68. doi:10.1016/0002-9343(86)90023-4.
155. Fukuda K, Namba M, Kawase I, Sakatani M, Ogura T, Kishimoto S, et al. [A case of sarcoidosis with multiple organ involvement including hypothalamic-pituitary dysfunction and diabetes insipidus]. Nihon Naika Gakkai Zasshi. 1985;74(11):1622–1627.
156. Ismail F, Miller JL, Kahn SE, Willcox PA. Hypothalamic-pituitary sarcoidosis: a case report. S Afr Med J. 1985 Jan 26;67(4):139–42. PubMed PMID: 3969566.
157. Vesely DL, Maldonodo A, Levey GS. Partial hypopituitarism and possible hypothalamic involvement in sarcoidosis: report of a case and review of the literature. Am J Med. 1977 Mar;62(3):425–31. doi:10.1016/0002-9343(77)90842-8.
158. Tang RA, Grotta JC, Lee KF, Lee YE. Chiasmal syndrome in sarcoidosis. Arch Ophthalmol. 1983 Jul;101(7):1069–73. doi:10.1001/archopht.1983.01040020071011.
159. Jackson A, Hood TR. Sarcoidosis with involvement of the pituitary gland. Ann Intern Med. 1958 Aug;49(2):467–71. doi:10.7326/0003-4819-49-2-467.
160. Morgan T, Coupland WG, Vanderfield GK, Church D. Hypothalamic-pituitary sarcoidosis. Australas Ann Med. 1965 Aug;14(3):250–6. doi:10.1111/imj.1965.14.3.250.
161. Garrett P, Langan C, Egan J, Mulloy E, O'Donnell J. Sarcoidosis, anterior pituitary failure, and diabetes insipidus. Ir J Med Sci. 1984 Mar;153(3):136-8. doi:10.1007/BF02939986.
162. Conn JW. Sarcoidosis Associated with Hypopituitarism. Br Med J. 1954 Dec 11;2(4901):1416.
163. Magnus EM. Two cases of sarcoidosis involving the hypophysis treated with corticotrophin and cortisone. Acta Endocrinol (Copenh). 1956 May;22(1):1–8. doi:10.1530/acta.0.0220001.
164. Higashida S, Akigawa S, Miura N, Sato T, Murota N, Tanoue M, et al. [A case of sarcoidosis complicated by diabetes insipidus, anterior pituitary dysfunction, and central alveolar hypoventilation]. Nihon Naika Gakkai Zasshi. 1990;79(7):954–955.
165. Al-Hussaini A, Al-Otaibi F, Al-Shammari M. Pituitary Sarcoidosis: A Rare Cause of Secondary Amenorrhea. J Endocr Soc. 2019 May 3;3(Suppl 1):A724. doi:10.1210/jendso/bvab048.1262.
166. Liu E, Rohr A, AlMehthel M. Neurosarcoidosis presenting with hypopituitarism. BMJ Case Rep. 2020 Oct 4;13(10):e235077. doi:10.1136/bcr-2020-235077 PubMed PMID: 33019535.
